# The dead, hardened floral bracts of dispersal units of wild wheat function as storage for active hydrolases and in enhancing seedling vigor

**DOI:** 10.1371/journal.pone.0177537

**Published:** 2017-05-11

**Authors:** Buzi Raviv, Gila Granot, Vered Chalifa-Caspi, Gideon Grafi

**Affiliations:** 1 French Associates Institute of Agriculture and Biotechnology of Drylands, The Institutes for Desert Research, Ben-Gurion University of the Negev, Midreshet Ben-Gurion, Israel; 2 The National Institute for Biotechnology, Ben-Gurion University of the Negev, Beer-Sheva, Israel; University of Illinois at Urbana-Champaign, UNITED STATES

## Abstract

It is commonly assumed that the dead, hardened floral bracts of the dispersal unit of grasses have been evolved to protect seeds from predation and / or assist in fruit/caryopsis dispersal. While these structures have important agronomical and economical implications, their adaptive value has not been fully explored. We investigated the hypothesis that the maternally derived hardened floral bracts have been evolved not just as a means for caryopsis protection and dispersal, but also as storage for substances that might affect seed germination and seedling vigor. Dead glumes as well as lemmas and paleas of wild emmer wheat (*Triticum turgidum var dicoccoides*) were found to store and release upon hydration active hydrolases including nucleases and chitinases. High nuclease activity was released upon hydration from glumes derived from wild strains of wheat including *Triticum urartu* and wild emmer wheat, while very low nuclease activity was detected in glumes derived from domesticated, free-threshing wheat cultivars (e.g., durum wheat). Germination from the intact dispersal unit of wild emmer wheat was delayed, but post germination growth was better than those of separated caryopses. Most notable was a significant increase in lateral root production on seedlings germinated from the intact dispersal unit. Proteome analysis of wild emmer wheat glumes revealed many proteins stored and released upon hydration including S1-type nucleases, peptidases, antifungal hydrolases such as chitinases and β-1,3-glucanase as well as pectin acetylesterase, a protein involved in cell wall degradation and remodeling. Also, reactive oxygen species (ROS)-detoxifying enzymes such as superoxide dismutase and ascorbate peroxidase were overrepresented in dead glumes of wild emmer wheat. Thus our study highlighted previously unknown features of the dispersal unit in wild wheat in which the dead, hardened floral bracts enclosing the caryopsis store active hydrolases and nutritional elements and probably growth promoting substances that facilitate seed longevity and germination and increase seedling vigor.

## Introduction

Flowering plants (angiosperms) represent the most diverse group of land plants. They have evolved a plethora of strategies to increase fitness through extensive modification of their reproductive organs, influorescence and flower parts [1]. Species in the grass family (Poaceae), particularly wheat, rice and maize provide a major staple food for human and are agricultural and economical most important crops. This family has evolved unique, modified influorescences and structures surrounding the flowers, which are commonly arranged in spikelets, each may contain one or multiple flowers [2]. A spikelet is composed of two hardened bracts at the base called glumes, which enclosed the flowers. The flower is commonly hermaphroditic, wind pollinated, and consists of external (lemma) and internal (palea) bracts. The perianth is reduced to two scales, called lodicules located at the base of the ovary; these scales expand at anthesis to allow for the spread of the lemma and palea and for pollination to occur. The fruit is a caryopsis composed of one seed in which the seed coat is fused with the pericarp.

Because the structures enclosing the caryopsis have important agronomical and economical implications, the domestication of wheat has occurred via genetic mutations that resulted in types of wheats with non-fragile rachises, soft glumes, and free-threshing caryopses [3,4]. *Triticum urartu* is the progenitor of the AA genome of wild emmer wheat (*Triticum turgidun* ssp. *dicoccoides*; genomes BBAA^u^) as well as of the domesticated durum wheat (*Triticum turgidum* subsp. *durum*, BBAA^u^), a tetraploid wheat derived from wild emmer wheat [5]. It is widely accepted that bread wheat (*Triticum aestivum* AABBDD) was originated via hybridization of hulled tetraploid emmer wheat with *Aegilops tauschii* (genomes DD) resulting in hexaploid spelt wheat (*Triticum aestivum* ssp. *spelta*, genomes BBAADD), from which free-threshing wheat evolved by mutations [6]. In spelt, like in wild emmer wheat and *Ae*. *tauschii*, glumes are tenaciously enclosed the caryopses and their liberation often requires a strong mechanical force.

The dispersal unit of wild wheat is composed of the spikelet whereby the caryopsis is enclosed by dead, hardened parts of glumes, lemmas that carry owns and paleas. Tenacious glumes and other hardened floral bracts (e.g., lemmas and paleas) of grasses are believed to function in seed dispersal and provide a physical shield protecting the seed from predation [7]. The awns of wild emmer wheat were shown to function as a motor fueled by daily humidity cycles that drive the dispersal unit into the ground [8]. This hygroscopic movement is possible due to the unique orientation of the cellulose fibrils that build the cell wall [9]. In addition, the glumes and hull of *Ae*. *kotschyi* were shown to contain germination inhibitors that appear to function *via* controlling GA (gibberelin) metabolism [10]. Yet, the function and the adaptive value of the dead, hardened floral bracts enclosing the caryopsis have not been fully explored.

We investigated the hypothesis that the maternally-derived dead, hardened floral bracts of wild emmer wheat dispersal unit have been evolved not just as a means for caryopsis protection and dispersal but also as a storage for substances that might affect seed germination and seedling vigor. Our study provide novel insight on the function of the dead, hardened floral bracts enclosing the caryopsis showing it serves as a storage for active hydrolases and growth promoting substances that can increase seedling vigor and consequently establishment and survival in the niche.

## Materials and methods

### Collection of dispersal units of Poaceae species

Dispersal units of wild emmer wheat (*Triticum dicoccoides* Köern.) and winter oat *Avena sterilis* were collected from their natural habitat in Israel, namely, Tabigha, Sea of Galilee on basalt soil (35°53’53” N 35°33’14” E) and road sides near Sede Boqer, in central Negev (34°47’20” N 34°51’01” E), respectively. Spikes of cultivated and wild strains of wheat grown in a net house were donated by Peleg Z. including *T*. *aurartu*, *T*. *monococcum*, *T*. *timopheevii*, *T*. *aestivum* and *T*. *durum*. All spikes were collected after they matured and stored at room temp for 1–2 years until used.

### In-gel nuclease assay and Peptide-N-glycosidase F (PNGase F) reaction

In gel nuclease assay was performed essentially as described [11] in polyacrylamide gel containing 300 μg/ml denatured salmon sperm DNA. Briefy, 10 mg of caryopses or ground floral bracts (glumes, lemmas and paleas) were incubated in 100 μl of phosphate-buffered saline (PBS) at 4°C for 8–12 h after which samples were centrifuged (4°C, 14,000 rpm, 5 min) and the supernatant was collected and used immediately or stored at -20°C until used. For in gel nuclease assay, 20 μl of the supernatant derived from caryopses or various floral bracts were incubated with sample buffer containing 2% SDS, 62.5mM Tris pH 6.8 and 10% glycerol and bromophenol blue for 1h at 37°C followed by separation on SDS/PAGE. The gel was washed twice, each time for 30 min, at room temp in buffer containing 10 mM Tris-HCl pH 7.5 and 25% isopropanol, followed by washing twice 15 min each with 10 mM Tris-HCl pH 7.5. Nuclease activity was performed by incubating the gel with 10 mM Tris-HCl pH 7.5 containing divalent cations (10 mM MgSO_4_, 10 mM CaCl_2_) for 75 min at 37°C. The gel was stained for 60–80 minutes with 10 mM Tris HCl pH 7.5 containing 2 μg/ml ethidium bromide and inspected under UV light.

Peptide -*N*-Glycosidase F (PNGase F, purchased from New England BioLabs) reactions were performed at 37°C for 2h in G7 Reaction Buffer according to the manufacturers’ protocol except that treatment with glycoprotein denaturing buffer was omitted.

### In-gel chitinase assay

In gel chitinase assay was performed essentially as described [12]. Briefly, 10 mg of caryopses or ground floral bracts (glumes, lemmas and paleas) were incubated in 100 μl 0.1M NaHPO_4_ (pH = 6) at 4°C for 8–12 h after which samples were centrifuged (4°C, 14,000 rpm, 5 min) and the supernatant was collected and used immediately or stored at -20°C until used. For in gel chitinase assay, 20 μl of the supernatant derived from caryopses or various floral bracts were first incubated in chitin sample buffer (15% sucrose, 2.5% SDS, 12.5 mM Tris-HCl pH 6.7, 0.01% Bromophenol Blue) for 1 h at 37°C and followed by separation on 12% SDS/PAGE containing 0.01% glycol chitin. The gel was incubated in buffer containing 100 mM sodium acetate (pH = 5.2) and 1% triton x-100 for 2 h at 37°C followed by staining for 5 min with 0.01% calcofluor white in 500 mM Tris-HCl (pH = 8.9). The gel was washed with distilled water for 1 h and visualized by UV transillumination.

### Germination assays

The germination assay was based on the ‘cigar roll’ method [13]. Ten uniform-size seeds or ten dispersal units of each genotype were placed on moist germination paper (25 cm × 38 cm; Anchor Paper Co., St. Paul, MN, USA), about 4 cm apart, 4 cm below the edge of the paper, with germ end down. The paper was covered with another sheet of moist germination paper and the sandwich was rolled to a final diameter of 2 cm. The base of each roll was placed in a glass beaker containing distilled water for three weeks. The length of the shoot organs was measured with a ruler; Leaf length was defined as the distance from the tip of the coleoptile to the tip of the first leaf. Images of the seedlings and roots were analyzed with ImageJ software. Then, the seedlings were dried in 60°C oven for two days and dry weight was measured. Statistical analysis of data was performed by two-tailed, unpaired t test using Graphpad software (https://www.graphpad.com/quickcalcs/).

### Nutrient analysis

For nutrient analysis, four dispersal units of wild emmer wheat were dissected into caryopses and floral bracts, namely, glumes, lemmas and paleas (GLPs) followed by incubation in 600 μl of ultrapure water (Milli-Q) for 12 hours on a cooled orbital shaker (4°C). Samples were centrifuged (14,000 rpm, 4°C) and the supernatant was filtered (0.22 μm spin filter) and diluted with 4.8 ml of Milli-Q water and subjected to nutrient analysis. Three measurements were performed for each chemical element. The content of K, P, Ca, Mg, S and Zn released upon hydration from wild emmer wheat dispersal unit was determined by inductively coupled plasma-optical emission spectroscopy (ICP-OES) using ICP-720-ES (Varian Inc., USA). Soluble anions were detected and identified by Ion chromatography (IC). IC was performed by ICS-5000 instrument (Dionex, Thermo Fisher Scientific). Obtained data were analyzed by Chromeleon 6.8 chromatography data system (Dionex, Thermo Fisher Scientific).

### Proteome analysis

Ground glumes (10 mg) derived from different dispersal units (three biological replicates) of wild emmer wheat grown in a greenhouse at were incubated in 100 μl of PBS at 4°C for 12 h followed by centrifugation (14,000 rpm, 4°C, 10 min). The supernatant containing proteins released from glumes was collected and subjected to proteomic analysis at The Smoler Protein Research Center at the Technion, Israel. Proteins released from glumes were digested with trypsin followed by separation and mass measurement by LC-MS/MS on Q-Exactive (Thermo) and identification by Discoverer 1.4 (with the search algorithm: Sequest; Thermo) against Triticum from NCBI and Triticum from Uniprot databases and against Uniprot unspecific.

In the Uniprot unspecific search we identified only proteins from *Triticum* and none from fungi. All the identified peptides were filtered with high confidence, top rank, mass accuracy, and a minimum of 2 peptides. High confidence peptides were passed the 1% FDR threshold (FDR = false discovery rate, is the estimated fraction of false positives in a list of peptides). Semi-quantitation was done by calculating the peak area of each peptide. The area of the protein was calculated from the average of the three most intense peptides from each protein. Three replicates were performed for the analysis of wild emmer wheat glume proteins.

A more stringent filtering was applied to the proteins, which are considered present in each plant. Considering that the average amino acid length of the proteins in the dataset was nearly 400 and assuming that 2 peptides are the minimum requirement for an average protein, we extrapolated this cutoff to consider protein length as the following: The minimum number of peptides per protein is the protein length divided by 200, but not less than 2 peptides. Additionally, the protein coverage by peptides from all samples must be higher than 30%. A protein is regarded as “present” in a plant if it is “present” in at least two replicates.

Functional annotation and enrichment analysis were performed as follows: *T*. *aestivum* protein names, their InterPro domains and gene ontology (GO) annotations were retrieved from UniProt. GO, GO slim and GO slim plant ontology files (in.obo format) were downloaded from the Gene Ontology web site (http://geneontology.org/page/download-ontology). GO enrichment analysis was carried out using BiNGO (v3.0.3) in Cytoscape (v3.4.0). All *T*. *aestivum* proteins from UniProt were used as the reference set. Over-representation analysis was performed using the hyper-geometric test, and the Benjamini-Hochberg FDR method was used for multiple testing correction. Proteome parameter definition is given in S4 Table.

## Results

### The dead, hardened organs of the dispersal unit of wild emmer wheat store and release upon hydration active hydrolases

**Fig 1:**
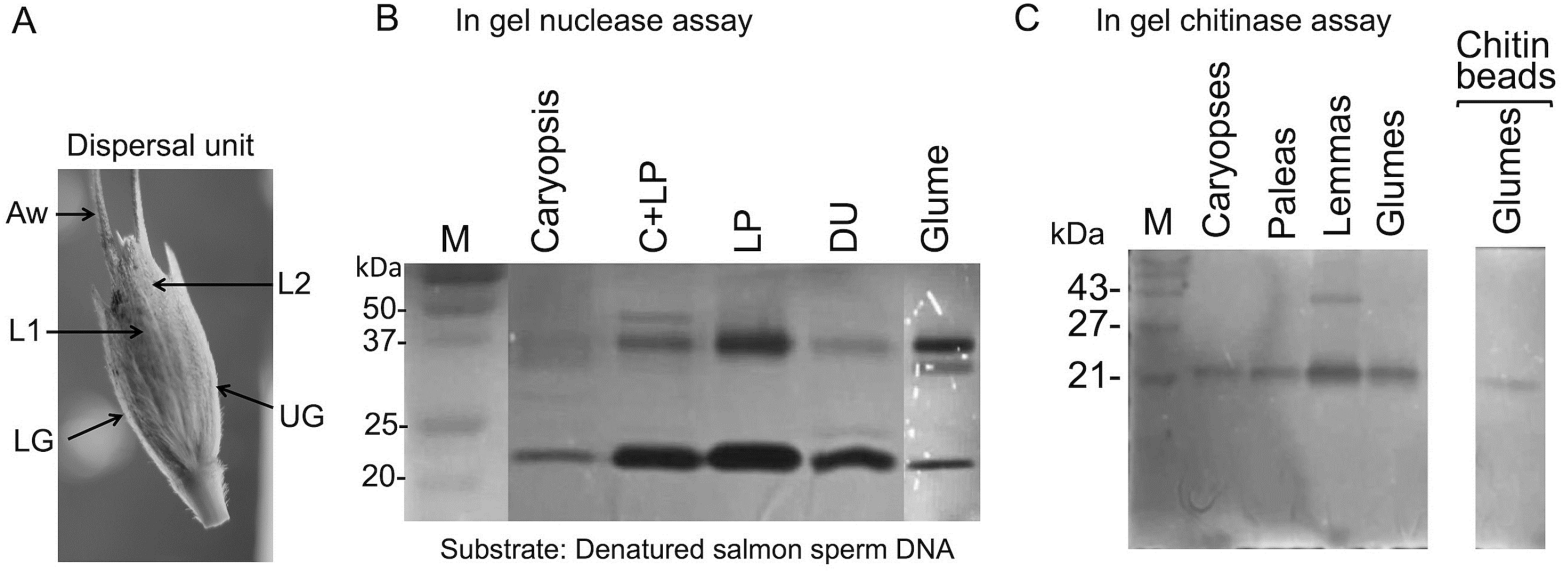
The hardened parts of wild emmer wheat dispersal unit possess active hydrolases. A, Dispersal units of wild emmer wheat. LG, lower glume; UG, upper glume; L1 and L2 lemmas enclosing floret 1 and 2, respectively; Aw, awn. B, In gel nuclease assay. Dispersal units of wild emmer wheat as well as the separated floral bracts were hydrated and released proteins were subjected to in gel nuclease assays using denatured salmon sperm DNA as substrate. C+LP, caryopsis with lemma and palea; DU, intact dispersal unit. C, In gel chitinase assay. Chitinases are released from the dead floral bracts of the dispersal unit of wild emmer wheat. Right panel, Chitinase released from glumes can be pulled down by chitin beads. DU, dispersal unit; LP, lemma and palea. M indicates protein molecular weight markers.

Dispersal units of wild emmer wheat collected in the field (Fig 1A) were separated into glumes, lemmas and paleas (GLPs) and caryopses, hydrated with PBS and substances released were collected and analyzed by in gel assays for hydrolase activities. In gel nuclease assay (Fig 1B), using denatured salmon sperm DNA as substrate revealed that all parts of the dispersal unit possess nuclease activities migrating in the gel to positions of about 35 (notable in glumes), 37 and 23 kDa. In gel chitinase assays revealed active chitinase at position of about 22 kDa, which is released from all parts of the dispersal unit including caryopses, glumes, lemmas and paleas (Fig 1C). Lemmas also released chitinase that migrated to position of about 40 kDa. The 22 kDa chitinase from glumes could be purified on chitin beads (Fig 1C, right panel) further verifying chitinase activity. These results demonstrate that dead, hardened floral bracts enclosing the caryopsis are not just passive entities involved in caryopsis protection and dispersal but rather an active unit that store and release upon hydration active hydrolases that may increase survival rate of germinating seeds.

### Nucleases are significantly reduced in extracts derived from hull of domesticated wheat

**Fig 2:**
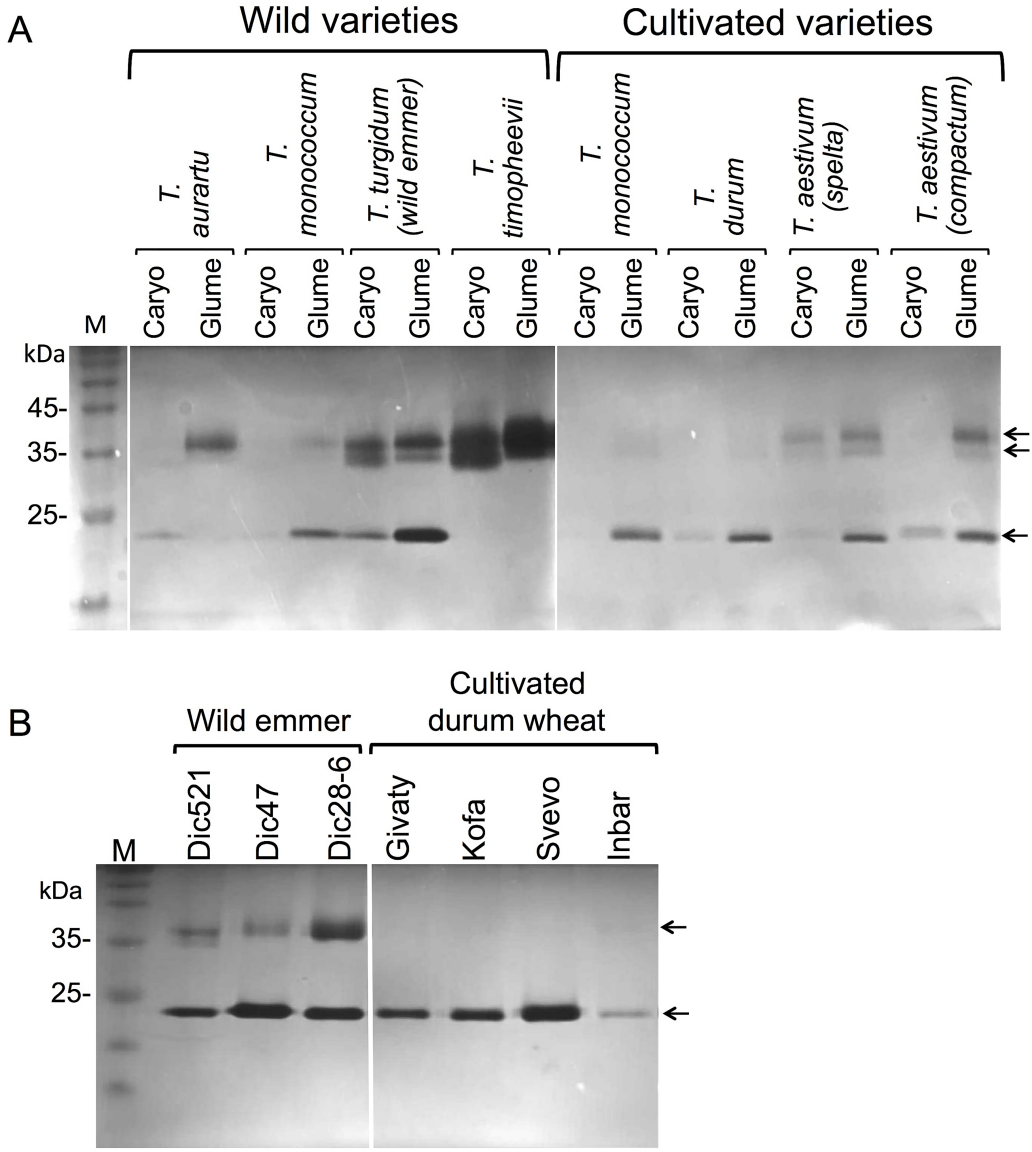
Glumes of domesticated, free trashing wheat have reduced nuclease activities. A, Intact caryopses and glumes derived from 3 spikelets of the indicated net-house-grown wild and cultivated varieties of wheat were hydrated (100 μl PBS) at 4°C for 12 h and half of the sup volume was collected and subjected to in-gel nuclease assay using denatured salmon sperm DNA as substrate. B, Comparison of nuclease activities in glumes derived from the indicated wild emmer wheat ecotypes with those derived from domesticated durum wheat cultivars (all were grown under a net-house conditions). Arrows indicate the positions of nucleases. M, protein molecular weight markers.

The domestication of wild wheat resulted in multiple cultivars with non-fragile rachises, soft glumes and free-threshing caryopses. We wanted to examine the proposition that some features characteristic of glumes of wild *Triticum* species may have been lost in the course of domestication. Glumes and caryopses were obtained from spikelets collected from a net house-grown wild and cultivated varieties of wheats including diploid wheat *Triticum urartu* (AA^u^) and *T*. *monococcum* (AA^m^), tetraploid wild emmer wheat (BBAA^u^) and *T*. *timopheevii* (GGAA^m^), the domesticated tetraploid durum wheat (BBAA^u^, derived from wild emmer wheat) and two cultivated hexaploid wheat, *T*. *aestivum* (spelta) and *T*. *aestivum* (compactum). Intact caryopses and chopped glumes (derived from 3 spikelets) were incubated with PBS at 4°C and equal volume of extracts were analyzed for nuclease activities using in gel assays. In gel nuclease assays showed that all examined wheat lines possess various nuclease activities at positions of about 23, 35 and 37 kDa, which were mainly released from both caryopses and glumes in wild wheat species; in cultivated varieties nucleases were released mainly from glumes showing significant reduction in activity of 35 and 37 kDa nucleases compared to wild wheat varieties (Fig 2A). Further comparison of glumes obtained from various net-house grown wild emmer wheat ecotypes with various net-house grown durum wheat cultivars clearly showed (Fig 2B) that all domesticated durum cultivars have lost the 35 and 37 kDa nucleases while retaining the 23 kDa nuclease activity.

**Fig 3:**
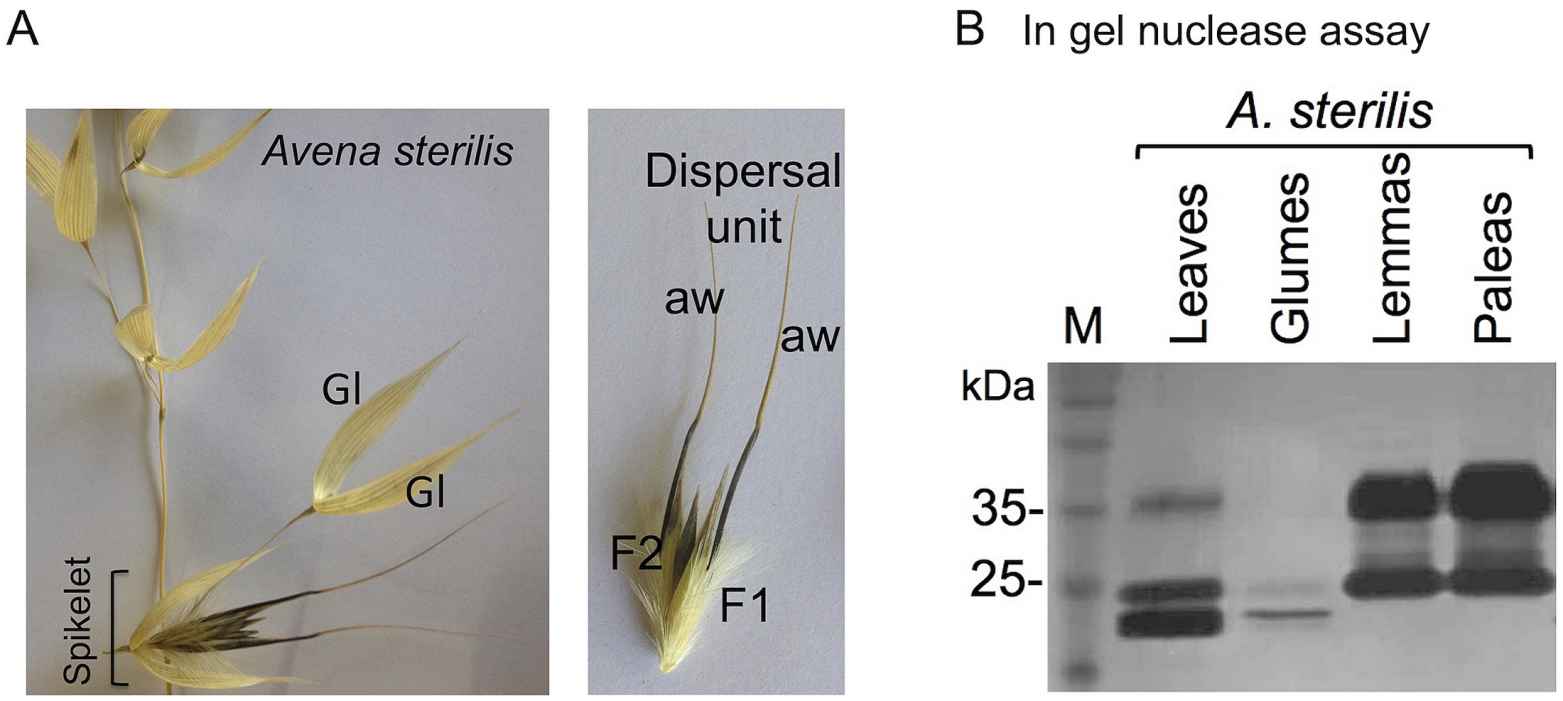
High levels of nuclease activity in the hardened parts of *A*. *sterilis* dispersal unit. A, Spikelet and dispersal unit of *A*. *sterilis*. Note the dispersal unit is composed of florets; glumes are left on the mother plant. aw, awn; F1 and F2, floret 1 and 2; Gl, glume. B, In gel nuclease assay of proteins released from leaves and the dead organs of the spikelet including glumes, lemmas and paleas. Denatured salmon sperm DNA was used as substrate. Note that very low nuclease activity was recovered in glumes.

The finding that durum wheat whose domestication resulted in free thrashing caryopses lost certain biochemical features of the glumes prompted us to investigate other Poaceae species whose dispersal unit is lacking glumes. We selected *Avena sterilis* L. (wild oat) whose dispersal unit is composed of florets (the rachilla disarticulate below the lowermost floret only), namely, caryopses enclosed by lemmas and paleas (Fig 3A). Interestingly, similarly to durum wheat glumes, wild oat glumes, which are left on the mother plant and are not included in the dispersal unit, showed very low nuclease activity compared to lemmas and paleas, particularly the loss of the 36 kDa nuclease activity (Fig 3B).

### Nucleases released from glumes upon hydration display S1-type endonuclease activity

**Fig 4:**
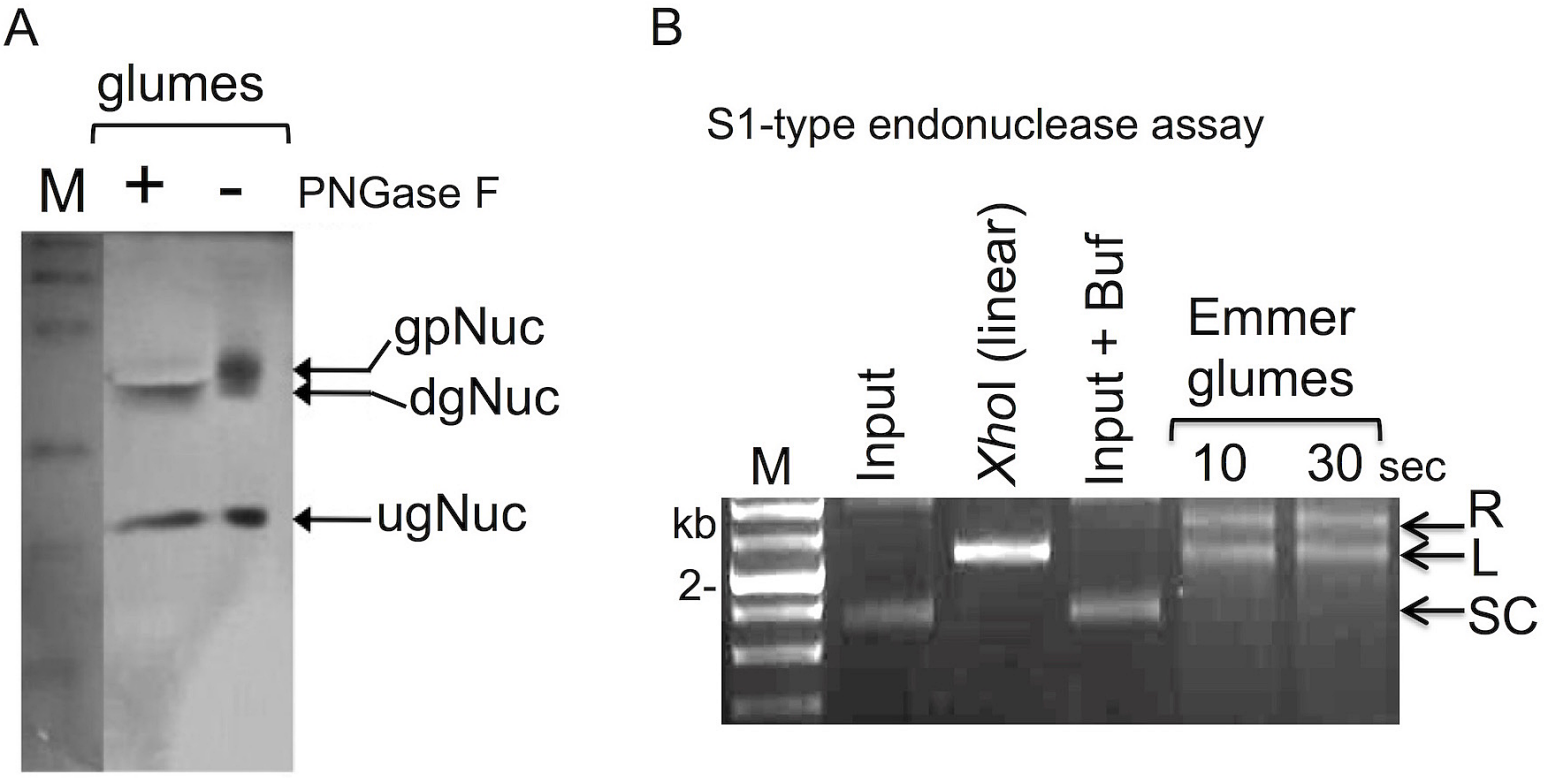
Nucleases released from glumes of wild emmer wheat are glycosylated and possess S1-type endonuclease activity. (A) proteins released from glumes following hydration were subjected to deglycosylation using PNGase F followed by in gel nuclease assay using denatured salmon sperm DNA as substrate. gpNuc, glycosylated nuclease; dgNuc, deglycosylated nuclease; ugNuc, unglycosylated nuclease. Note, that PNGase F treatment resulted in a mobility shift but did not affect nuclease activity. (B) In vitro endonuclease assay. Supercoiled plasmid DNA (1 μg) was incubated for the indicated time points at room temperature with proteins released from glumes of wild emmer wheat and reactions were stopped by adding chelating agent EDTA to 50 mM. The positions of the different topological forms of plasmid DNA are indicated: R, relaxed form; L, linear; SC, supercoiled plasmid DNA. *Xho*I is the linear plasmid generated by restriction; M, molecular size markers of 1 kb DNA ladder.

A 36 kDa nuclease activity was recovered in glume extracts by in gel nuclease assays using either denatured salmon sperm DNA or yeast torula RNAs (not shown) leading to the hypothesis that this activity may be related to S1/P1 type endonucleases. First, S1/P1 type endonucleases are bifunctional capable of cleaving both RNA and single stranded DNA and second the 36 kDa nuclease released from glumes has nearly the predicted molecular mass of S1-type endonucleases identified in various monocots species including *Triticum aestivum* (accession no: CDM80640), *Brachypodium distachyon* (XP_003567956), *Hordeum vulgare* (BAJ95618), *Oryza sativa* (XP_015614478) and *Zea mays* (ACG43533). The S1/P1 type endonucleases were shown to undergo N-glycosylation that may affect their subcellular localization and activity [14,15]. We first examined the glycosylation status and its importance for endonuclease activity released from glumes of wild emmer wheat using Peptide-N-Glycosidase F (PNGase F) that cleaves N-linked glycans from glycoproteins. This assay showed (Fig 4A) that the 22 kDa nuclease is essentially not N-glycosylated (ugNuc) as no change in its mobility was observed following PNGase F treatment. However, treatment with PNGase F affected the mobility of the 36 kDa protein but did not abolish nuclease activity. Accordingly, the 36 kDa nuclease was disappeared following PNGase F treatment and a notable activity was observed at position of 34 kDa. This suggests that either glycosylation is not required for activity or that the limited action of PNGase F on glycoproteins [16] resulted in nuclease decorated with sugars that cannot be removed by PNGase F and might confer nuclease activity.

S1-type endonucleases, such as *Aspegillus* S1 nuclease and mung bean nuclease are widely used in molecular biology applications and although their preferred substrate is single stranded nucleic acids they are capable of introducing nicks and double strand DNA breaks (DSBs) into supercoiled plasmid DNA converting it to relaxed and/or linear forms—a well established method for monitoring single-stranded DNA endonuclease activity [17]. Single-stranded DNA endonucleases can change the topology of double-stranded supercoiled plasmid DNA as a result of torsional strain within superhelical DNA resulting in local denaturation that can be targeted by S1-type endonucleases [18]. To examine for S1-type endonuclease activity we incubated supercoiled plasmid DNA with proteins extracted from glumes of wild emmer wheat for short time periods in a buffer containing Ca^2+^/Mg^2+^ cations as cofactors. Indeed nucleases released from glumes of emmer wheat were capable of converting supercoiled plasmid DNA (SC) into relaxed (R) and linear (L) forms (Fig 4B). Thus, nucleases extracted from glumes contain S1 type endonucleases capable of introducing nicks and double strand DNA breaks into superhelical DNA.

### Seedling vigor is enhanced when germinating from the intact dispersal unit

**Fig 5:**
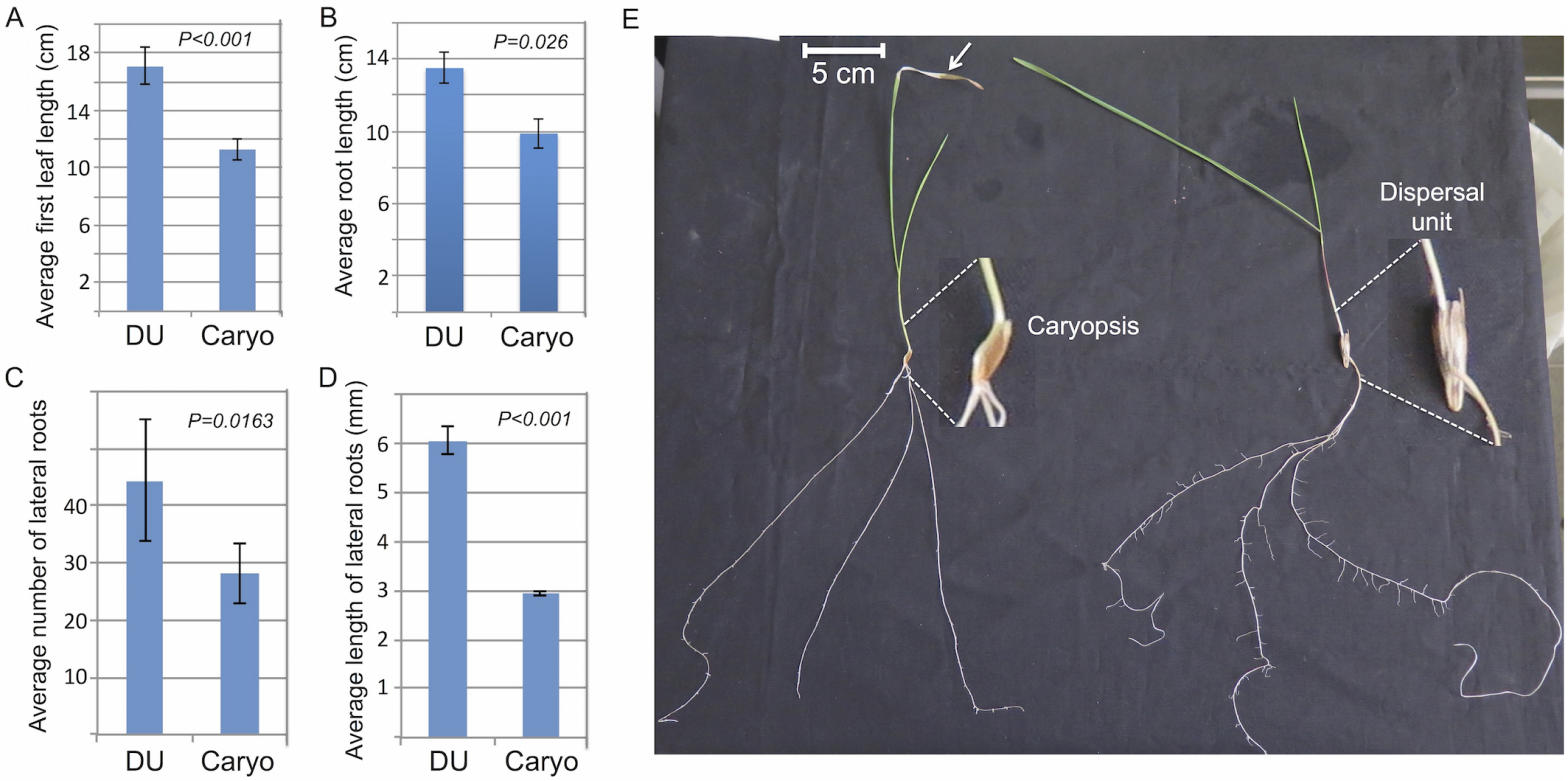
Germination from the intact dispersal unit increases seedling vigor. Dispersal unit (DU) or caryopses (Caryo) of wild emmer wheat were germinated using the ‘cigar roll’ method and the average length of first leaf (A), primary roots (B), number of lateral roots (C) and the average length of lateral roots (D) were recorded after 4 weeks. Bars represent the standard error (SE). Differences are statistically significant as determined by two-tailed P value using unpaired t test with Graphpad software. (E) Increase number and length of lateral roots on seedlings germinated from the intact dispersal unit. Magnified caryopsis and dispersal unit are indicated by broken lines. Arrow points to accelerated senescence of the tip of the first leaf derived from germinated caryopsis. The results are the sum of four biological experiments.

To assess the biological significance of germination from the dispersal unit, we germinated wild emmer wheat either as a naked caryopsis where lemmas, paleas and glumes were removed or as intact dispersal unit. We used the ‘cigar roll’ system on standard germination papers soaked in distilled water and placed in a growth room. Notably, the caryopsis alone was germinated faster than the intact dispersal unit with a delay of about 4 days. However, after germination, seedlings that emerged from the dispersal unit grew and developed better than seedlings that emerged from the caryopsis alone. Accordingly, DU seedlings had higher average length of the first leaf (Fig 5A) and of the primary roots (Fig 5B) as well as significantly higher lateral root formation (Fig 5C) and growth (Fig 5D and 5E). Furthermore, seedlings that emerged from the caryopsis alone displayed accelerated senescence of the tip of the first leaf compared to DU seedlings (see arrow Fig 5E). Thus it appears that the dispersal unit components provide beneficial substances that enhanced seedling vigor.

### The hardened parts enclosing the caryopsis store and release high levels of nutrients upon hydration

**Fig 6:**
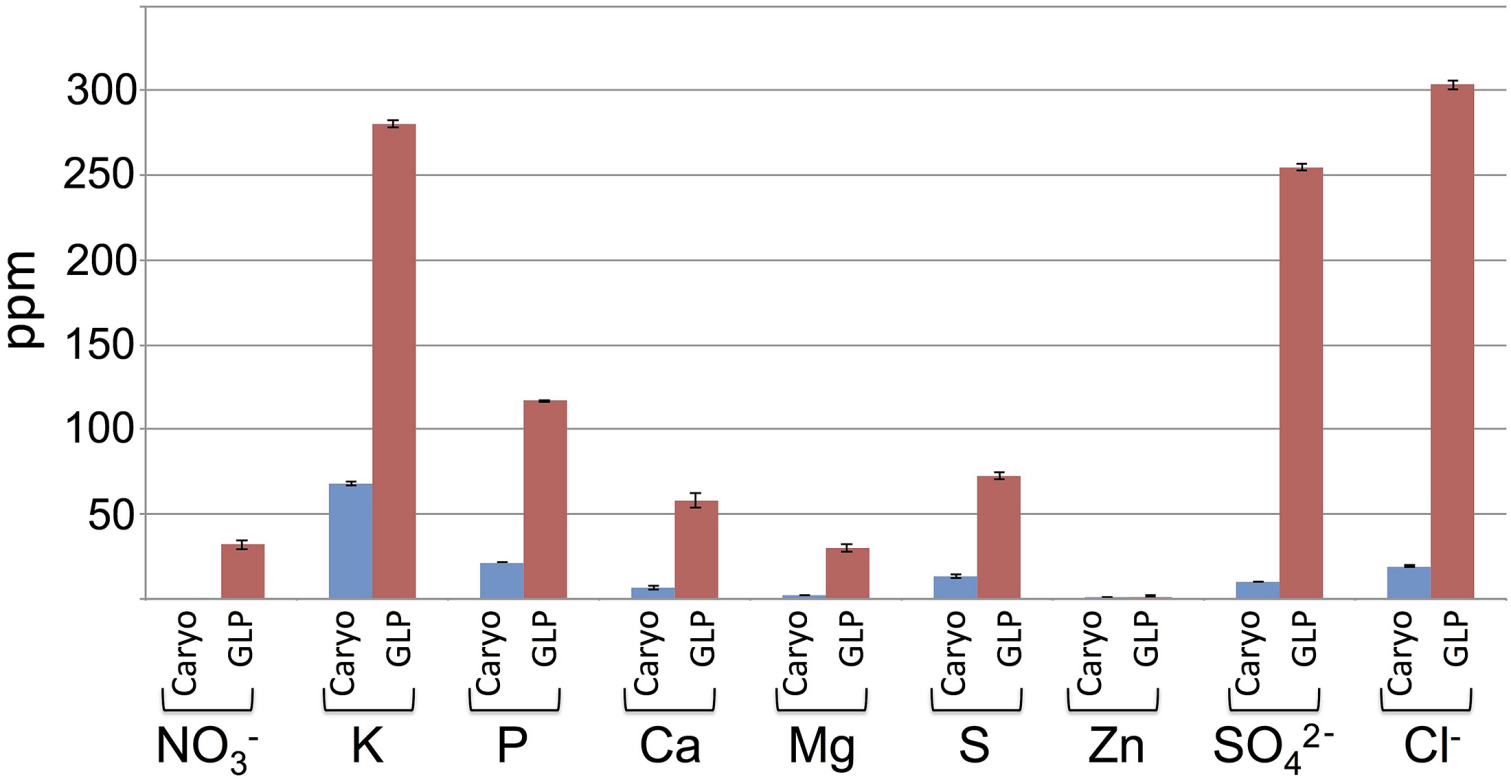
High levels of nutrients are stored and released from glumes, lemmas and paleas (GLPs) upon hydration. Intact caryopses (Caryo) and hardened bracts enclosing the caryopsis (GLPs) were incubated in 0.8 ml PBS at 4°C for 12 h, the sup was collected and subjected to nutrient detection by ICP-OES and to anion detection by Ion chromatography. The concentrations (ppm) of each element detected in extract derived from caryopses and GLPs are shown. Bars represent the standard error.

The finding that seedlings derived from intact DU perform better than seedlings derived from the caryopsis suggests that the dead bracts enclosing the caryopsis (glumes, lemmas and paleas) contribute substances such as nutrients that promote seedling vigor. Nutrient composition and content released from dead bracts of the DU were compared with those released from the caryopsis. Accordingly, four DUs of wild emmer wheat were separated into caryopses (Caryo) and glumes, lemmas and paleas (GLPs) and incubated in 0.8 ml of PBS at 4°C for 12 h, the sup was collected and analyzed by ICP-OES for detection of micro- and macro-elements. The results showed (Fig 6) that GLPs released high levels of macroelements compared to the caryopses. Accordingly, GLPs were rich particularly in potassium (K) but also in phosphorus (P), calcium (Ca), magnesium (Mg) and sulfur (S) relative to the caryopses. Further analysis of anions revealed that GLPs released high levels of sulfate (SO_4_^2-^) and chloride (Cl^-^) but also nitrate (NO_3_^-^), while very low levels were released from caryopses following hydration.

### Proteome analysis revealed hundreds of proteins stored in dead glumes of wild emmer wheat

**Fig 7:**
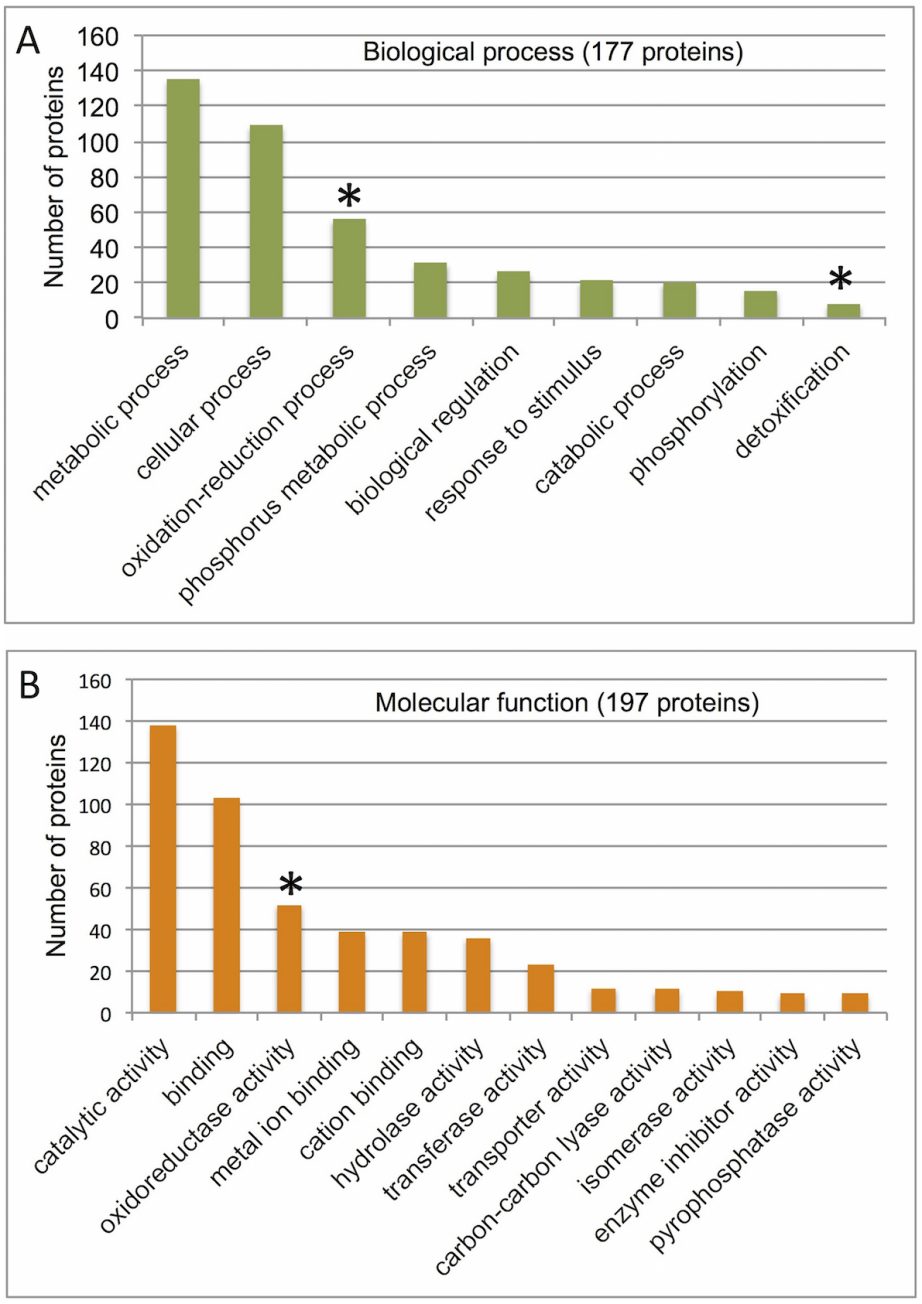
Analysis of proteins released from the dead glumes of wild emmer wheat. Proteome data were analyzed for proteins released from dead glumes of wild emmer wheat (mature dispersal units were collected at 2015 and analyzed during February 2017; three biological replicates). (A and B) Selected GO categorization for biological process and molecular function, respectively. Asterisks in A and B marked overrepresented protein group compared to frequency in the wheat genome. In brackets, the number of proteins identified for the examined category.

Proteome analysis was performed by trypsin digestion of proteins released from the dead glumes of wild emmer wheat (SG-Bas ecotype) followed by separation on LC-MS/MS on LTQ-Orbitrap and identification by Discoverer software against the *Triticum* from Uniprot databases (S1 Table). Notably, in the Uniprot unspecific search we identified only proteins from *Triticum* and none from fungi. Implementing the cutoffs described in Material and methods, 255 proteins were identified (S2 Table). Functional categorization of these proteins revealed (S3 Table) that among the 177 proteins recognized in biological process category, 135 proteins are involved in metabolic processes, 56 proteins are involved in oxidation-reduction processes and 21 proteins are related to response to stimuli (Fig 7A). Molecular function analysis (Fig 7B) showed that among the 197 proteins recognized in this category, 138 proteins have catalytic activity including 52 proteins with oxireductase activity and 36 proteins with hydrolase activity. Among hydrolases we identified chitinases, S1-type endonucleases, ribonucleases and peptidases. Interestingly, the proteome data revealed multiple ROS detoxifying enzymes such as Cu/Zn and Mn superoxide dismutases, plant ascorbate peroxidase as well as proteins containing glutathione-disulphide reductase and thiol specific antioxidant domains. The proteome data also uncovered pectin acetylesterase an enzyme that catalyzes deacetylation of pectin and is implicated in softening and loosening of the primary cell wall [19].

## Discussion

Angiosperm evolution has led to the development of diverse fruit morphologies, which is believed to assist seed dispersal [1]. In angiosperm, the dispersal unit is variable often composed of the seed (the embryo enclosed by the maternally derived seed coat), the seed with attached structure (e.g., the winterfat *Eurotia lanata*, Chenopodiaceae), the fruit (e.g., *Medicago* species; caryopsis in grasses) or the fruit with attached structures, such as the dispersal unit of various grasses, which are composed of the whole spikelet (wild emmer wheat) or the florets (*Avena sterilis*). Although the term dispersal unit highlights an entity that is specialized for dispersal it has been previously shown that the dispersal unit has evolved to fulfill multiple functions that contribute to seedling establishment including dispersal, embryo protection, seed positioning and orientation, seed fixation, water uptake and respiration [20]. Data presented here highlighted two aspects of the dispersal unit of Poaceae species, namely, *Triticum* and *Avena* species, which have not been recognized previously. These include the finding that dead floral bracts of the dispersal unit (glumes, lemmas and paleas) function as storage for active hydrolases that are released upon hydration to the ‘seedsphere’ and that seedling vigor is enhanced when germinated from the whole dispersal unit compared to the thrashed seed. In addition, out data showed that the dead floral bracts of the dispersal unit of wild emmer wheat function as a nutritional storage that might provide immediate supply of necessary nutrients at early stages of germination.

It is commonly believed that during programmed cell death (PCD), macromolecules such as proteins, DNA and RNA are degraded and their constituents remobilized into other parts of the plant [21]. This hypothesis is challenged by the data presented here showing that dead organs enclosing embryos in grasses store and release active hydrolases including nucleases and chitinases upon hydration. Proteome analysis of dead glumes revealed hundreds of proteins being stored and released upon hydration, including hydrolases, proteins related to oxidation reduction processes among them reactive oxygen species (ROS)-detoxifying enzymes as well as proteins related to stress response. Among hydrolases identified in the proteome data we identified S1-type endonucleases and chitinases further supporting functional analyses of glume extracts. In addition, we identified pathogenesis-related (PR) protein 1–1, PR-1-5 as well as PR-4 that encodes for RNase, as well as peptidases. Single stranded DNA endonucleases that are belong to the S1/P1 family of endonucleases are capable of introducing double strand DNA breaks into superhelical DNA [15,22]. Presently, we do not know the exact function of these nucleases for seed germination and seedling establishment. Generally, endonucleases have been implicated in diverse cellular processes including DNA synthesis and DNA repair [23] as well as in fragmentation of genomic DNA during PCD [15,24]. Because of their capability to induced DSBs, endonucleases may function as seed defense factors against plasmid-containing soil pathogens. In this respect, the *Clavibacter michiganensis subsp*. *michiganensis*, a Solanaceae species-pathogenic Gram-positive actinomycete contains two plasmids, designated pCM1 and pCM2, which are important for its pathogenic activity, inasmuch as plasmid-free derivative CMM100 can colonize tomato, but showing no disease symptoms [25]. Targeting these superhelical plasmids by endonucleases released from dead floral bracts enclosing the caryopsis can lead to neutralization of virulent genes and conversion of a potentially pathogen into a non-pathogenic one. Also, RNases were shown to inhibit growth of pathogenic fungi. Accordingly, exogenous application of S-like RNase NE into the extracellular space of leaves inhibits the development of *Phytophthora parasitica* [26,27], a oomycete soilborne pathogen with a wide range of host plants. In addition, the Wheatwin1 PR4 RNase, which was identified in our proteomic data, was shown to enter inside fungal cells without affecting the integrity of cell walls and possesses antifungal activity, which is dependent on its enzymatic activity [28]. In-gel RNase assays showed the activity of multiple RNAses released from the glumes (data not shown) highlighting the wide range of stored molecules that have the potential to act as pathogen inhibitors.

In gel assays also revealed active chitinases that could potentially act against pathogens. Chitinases are enzymes that degrade chitin an abundant polysaccharide found in a variety of organisms including insects, fungi, yeast, and algae. Chitinase and glucanase genes were often over-expressed in plants to confer resistance against fungal pathogens [29–31]. Thus, it appears that storage of hydrolases in maternally-derived glumes have been evolved, at least in part, to provide a defense mechanism for the germinating seed from soil-borne fungal pathogens. This hydrolase-based type of defense mechanism is weakened in glumes, which are no longer part of the dispersal unit as demonstrated in *Avena sterilis* and in domesticated, free-threshing durum wheat.

Overrepresentation of proteins involved in oxidation-reduction processes may demonstrate their importance in seed biology [32,33]. Generally, the seed antioxidant system plays an important role in scavenging ROS such as peroxides and superoxides to protect embryonic tissues from damages. Notably, ROS have also a signaling role, which is important for seed germination [34]. ROS can be neutralized by a variety of enzymes including superoxide dismutase (SOD) that catalyzes the dismutation of the superoxide (O_2_^−^) radical into either molecular oxygen (O2) or hydrogen peroxide (H_2_O_2_), as well as catalase and ascorbate peroxidase or thiol-based proxidases (PRXs) that detoxify H_2_O_2_ [33]. Interestingly, the proteome data uncovered several ROS detoxifying proteins including Cu/ZnSOD and MnSOD as well as alkyl hydroperoxide reductase subunit C/ thiol specific antioxidant (TSA) an important antioxidant that detoxifies sulphur-containing radicals. It is thus possible that dead organ enclosing the caryopsis has been evolved also to provide seeds and seedlings with a defense mechanism against the toxic effects of ROS. Indeed, ROS detoxifying mechanisms appears to play a key role in seed germination and seed storability [35].

Of particular interest was the biological effect of the dead hardened floral bracts on seedling vigor. In all germination experiments, the intact dispersal unit (DU) showed delay in germination time, that is, protrusion of roots, compared to the naked caryopsis. This is probably because the dead hardened floral bracts enclosing the caryopsis pose a physical barrier for root emergence. Indeed, the study of germination of various Brassicaceae species having indehiscent fruits suggested that the pericarp plays a dominant role in seed dormancy by mechanically restricting embryo growth [36]. Thus, to allow for radicle protrusion and seed germination, the hardened bracts might undergo weakening by cell wall degrading enzymes such as pectinolytic enzymes that hydrolyze pectic substances in plants [37] and contribute to degradation and re-modelling of plant cell walls [38]. We assume that such enzymes might be released upon hydration from the glumes, lemmas, paleas or from the caryopsis and assist in loosening and weakening of the caryopsis coat (fused seed coat and pericarp) as well as the hardened floral bracts that enclose the caryopsis to allow for root emergence. Indeed, the proteome data allows the identification of a pectin acetylesterase that is released from glumes upon hydration. This enzyme catalyzes the removal of acetyl esters from pectin backbone making it accessible to pectin-degrading enzymes, such as pectate lyases leading to softening and loosening of cell walls [19,39].

Notably, post germination growth and development were enhanced in seedlings derived from the intact DU. Particularly, seedlings derived from the DU have significantly higher number and higher length of lateral roots than seedlings derived from naked caryopsis. Similar effects were reported for germination of the winterfat dispersal unit, which consists of hairy bracts enveloping utricle (fruit) and the seed, which is composed of testa, embryo and perisperm (nutritive tissue made from the nucellus). Accordingly, the removal of the hairy bracts significantly reduced seedling establishment and vigor [40,41]. Enhanced production of lateral roots in seedlings derived from the intact DU suggests for the release of substances from the dead floral bracts that can promote lateral root production. In a variety of plant species, application of the phytohormone auxin was shown to induce lateral root formation from a subset of founder cells in the pericycle layer [42–44]. It is thus possible that induction of lateral root formation in seedlings derived from intact DU may be mediated by auxin, which is stored in and released from the dead, hardened floral bracts upon hydration, a topic currently studied in the lab. The notable increase in length of lateral roots might be related to nutritional effect brought about by substances released from the dead floral bracts namely, glumes, lemmas and paleas (GLPs). Indeed, the analysis of nutrient revealed high levels of potassium and phosphorus as well as sulfate and nitrate released from GLPs, which could explain, at least partly, the beneficial effect of the intact DU on lateral root growth. Early work with barley (*Hordeum* vulgare) showed that local supply of nitrate or phosphate stimulated lateral root growth [45]. Multiple reports highlighted nitrate as a major factor contributing to lateral root elongation [46,47]. Also, potassium might be required for lateral root growth inasmuch as potassium deficiency was reported to reduce length of lateral roots in tobacco seedlings probably *via* changing auxin distribution [48].

## Conclusions

The dispersal units of Poaceae species often composed of the maternally-derived dead, hardened floral bracts that enclose the caryopsis have been evolved not only for protecting caryopses from predation or as a means for seed dispersal and placement in the ground but also as an active entity that provide defense, growth regulatory factors and probably nourishing substances that assist germination and promote seedling vigor and consequently seedling survival in the niche. This appears to be a general phenomenon in plant seed biology whereby dead organs enclosing embryos including glumes in grasses and seed coats in dicotyledonous species such as Brassicaceae and Fabaceae species (S1 File) function as storage for active proteins and other substances that are released upon hydration and might improve seed persistence in soil, germination and seedling establishment. Interestingly, in wheat, some features appear to be lost from dead floral bracts during domestication that resulted in free-threshing seeds. The realization that dead organs enclosing embryos store active hydrolytic enzymes and proteins involved in reduction oxidation processes and possibly multiple beneficial substances might change the manner in which seeds are treated and stored in seed banks.

## Supporting information

S1 File Raviv et al. submitted manuscript. (PDF)

S1 Table Data set 1. Proteome raw data of all proteins released from dead glumes of wild emmer wheat.
(XLSX)

S2 Table Data set 2. List of proteins released from dead glumes of wild emmer wheat after filtering.
(XLSX)

S3 Table Data set 3. GO term classification.
(XLSX)

S4 Table Proteome parameter definition. (PDF)
