# Supplementary material for: The dead, hardened floral bracts of dispersal units of wild wheat function as storage for active hydrolases and in enhancing seedling vigor
Source: PLoS One. 2017 May 11;12(5):e0177537. doi: 10.1371/journal.pone.0177537 (PMC5426743; doi:10.1371/journal.pone.0177537)
Supplement: S1 File — (PDF) [file pone.0177537.s001.pdf]

PLOS ONE

Dead Organs Enclosing Embryos Function as Long-Term Storage for Active Hydrolytic Enzymes

--Manuscript Draft--

**Manuscript Number:**	
**Article Type:**	Research Article
**Full Title:**	Dead Organs Enclosing Embryos Function as Long-Term Storage for Active Hydrolytic Enzymes
**Short Title:**	Long-term storage of active proteins in dead organs enclosing embryos
**Corresponding Author:**	Gideon Grafi<br>Ben Gurion University<br>Midreshet Ben Gurion, ISRAEL
**Keywords:**	Anastatica hierochuntica; Antimicrobial substances; Arabidopsis thaliana; Chitinases; Dead seed coat; Dead organs enclosing embryo; Endonucleases; Hydrolases; Long-term storage; Sinapis alba; Seeds; Brassicaceae; Fabaceae; Proteome analysis; Mucilage
**Abstract:**	Seed development culminates in programmed cell death (PCD) and hardening of organs enclosing the embryo (e.g., pericarp, seed coat, endosperm) providing essentially a physical shield for protection during storage in the soil. It is commonly believed that during PCD macromolecules such as proteins are degraded and their constituents remobilized into other plant parts. We examined the proposal that dead organs enclosing embryos are unique entities that store and release upon hydration active proteins that might increase seed persistence in soil, germination and seedling establishment. Proteome analyses of dead seed coats of Brassicaceae species revealed hundreds of proteins being stored and released upon hydration many are stress-associated proteins such as nucleases, proteases and chitinases. Functional analysis of exalbuminous seeds (endosperm is consumed and undergoes death during maturation) of Brassicaceae and cotylespermous seeds (endosperm is short lived and cotyledons assume the function of storage tissue) of Fabaceae species revealed that dead seed coats and possibly remnants of the endosperm tissue function as long-term storage for multiple active hydrolytic enzymes (e.g., nucleases) that can persist in active forms for decades. Substances released from the dead seed coat of the annual desert plant Anastatica hierochuntica displayed strong antimicrobial activity. Our data highlighted a previously unrecognized feature of dead organs enclosing embryos (e.g., seed coat, endosperm) functioning not only as a physical shield for embryo protection but also as a long-term storage for active proteins and other substances that are released upon hydration to the "seedsphere" and could contribute to seed persistence in the soil, germination and seedling establishment.
**Order of Authors:**	Buzi Raviv
	Lusine Aghajanyan
	Gila Granot
	Vardit Makover
	Yitzchak Gutterman
	Omer Frenkel
	Gideon Grafi
**Opposed Reviewers:**	
**Additional Information:**	
**Question**	**Response**
**Financial Disclosure**	This work was supported by the Harbour Foundation to B.R. and by the Margolin foundation to G. Grafi.

Powered by Editorial Manager® and ProduXion Manager® from Aries Systems Corporation

Please describe all sources of funding that have supported your work. **This information is required for submission and will be published with your article, should it be accepted.** A complete funding statement should do the following:<br><br>Include **grant numbers and the URLs** of any funder's website. Use the full name, not acronyms, of funding institutions, and use initials to identify authors who received the funding.<br>**Describe the role** of any sponsors or funders in the study design, data collection and analysis, decision to publish, or preparation of the manuscript.<br>If the funders had **no role** in any of the above, include this sentence at the end of your statement: "*The funders had no role in study design, data collection and analysis, decision to publish, or preparation of the manuscript.*"<br><br>However, if the study was **unfunded**, please provide a statement that clearly indicates this, for example: "*The author(s) received no specific funding for this work.*"<br><br>* typeset	
**Competing Interests**<br><br>You are responsible for recognizing and disclosing on behalf of all authors any competing interest that could be perceived to bias their work, acknowledging all financial support and any other relevant financial or non-financial competing interests.<br><br>Do any authors of this manuscript have competing interests (as described in the PLOS Policy on Declaration and Evaluation of Competing Interests)?<br><br>**If yes**, please provide details about any and all competing interests in the box below. Your response should begin with this statement: *I have read the journal's policy and the authors of this manuscript have the following competing interests:*<br><br>**If no** authors have any competing interests to declare, please enter this	"The authors have declared that no competing interests exist."

statement in the box: "*The authors have declared that no competing interests exist.*"<br><br>* typeset	
**Ethics Statement**<br><br>You must provide an ethics statement if your study involved human participants, specimens or tissue samples, or vertebrate animals, embryos or tissues. All information entered here should **also be included in the Methods section** of your manuscript. Please write "N/A" if your study does not require an ethics statement.<br><br>**Human Subject Research (involved human participants and/or tissue)**<br><br>All research involving human participants must have been approved by the authors' Institutional Review Board (IRB) or an equivalent committee, and all clinical investigation must have been conducted according to the principles expressed in the Declaration of Helsinki. Informed consent, written or oral, should also have been obtained from the participants. If no consent was given, the reason must be explained (e.g. the data were analyzed anonymously) and reported. The form of consent (written/oral), or reason for lack of consent, should be indicated in the Methods section of your manuscript.<br><br>Please enter the name of the IRB or Ethics Committee that approved this study in the space below. Include the approval number and/or a statement indicating approval of this research.<br><br>**Animal Research (involved vertebrate animals, embryos or tissues)**<br><br>All animal work must have been conducted according to relevant national and international guidelines. If your study involved non-human primates, you must provide details regarding animal welfare and steps taken to ameliorate suffering; this is in accordance with the recommendations of the Weatherall report, "The use of non-human primates in research." The relevant guidelines	"N/A"

Powered by Editorial Manager® and ProduXion Manager® from Aries Systems Corporation

followed and the committee that approved the study should be identified in the ethics statement.<br><br>If anesthesia, euthanasia or any kind of animal sacrifice is part of the study, please include briefly in your statement which substances and/or methods were applied.<br><br>Please enter the name of your Institutional Animal Care and Use Committee (IACUC) or other relevant ethics board, and indicate whether they approved this research or granted a formal waiver of ethical approval. Also include an approval number if one was obtained.<br><br>**Field Permit**<br><br>Please indicate the name of the institution or the relevant body that granted permission.	
**Data Availability**<br><br>PLOS journals require authors to make all data underlying the findings described in their manuscript fully available, without restriction and from the time of publication, with only rare exceptions to address legal and ethical concerns (see the PLOS Data Policy and FAQ for further details). When submitting a manuscript, authors must provide a Data Availability Statement that describes where the data underlying their manuscript can be found.<br><br>Your answers to the following constitute your statement about data availability and will be included with the article in the event of publication. **Please note that simply stating 'data available on request from the author' is not acceptable.** ***If, however, your data are only available upon request from the author(s), you must answer "No" to the first question below, and explain your exceptional situation in the text box provided.***<br><br>Do the authors confirm that all data underlying the findings described in their manuscript are fully available without restriction?	Yes - all data are fully available without restriction
Please describe where your data may be found, writing in full sentences. **Your answers should be entered into the box below and will be published in the form**	"All relevant data are within the paper and its Supporting Information files."

**you provide them, if your manuscript is accepted.** If you are copying our sample text below, please ensure you replace any instances of XXX with the appropriate details.<br><br><br><br>If your data are all contained within the paper and/or Supporting Information files, please state this in your answer below. For example, "All relevant data are within the paper and its Supporting Information files."<br>If your data are held or will be held in a public repository, include URLs, accession numbers or DOIs. For example, "All XXX files are available from the XXX database (accession number(s) XXX, XXX)." If this information will only be available after acceptance, please indicate this by ticking the box below.<br>If neither of these applies but you are able to provide details of access elsewhere, with or without limitations, please do so in the box below. For example:<br><br>"Data are available from the XXX Institutional Data Access / Ethics Committee for researchers who meet the criteria for access to confidential data."<br><br>"Data are from the XXX study whose authors may be contacted at XXX."<br><br>* typeset	
Additional data availability information:	

Powered by Editorial Manager® and ProduXion Manager® from Aries Systems Corporation

Dear Editor,
Our manuscript entitled "**Dead Organs Enclosing Embryos Function as Long-Term Storage for Active Hydrolytic Enzymes**" by Raviv et al. provides novel insight into the function of the dead organs enclosing the embryo, which has not been well recognized previously. The work presented here continues our study on the biological significance of dead organ enclosing embryos in wild wheat (manuscript submitted to PLOS One, under revision), where we show that the dead, hardened floral bracts of the dispersal unit (i.e., glumes, lemmas and paleas) function as a storage for active hydrolases and in enhancing seedling vigor. Here we show that the dead organs enclosing embryos in dicots function not just as a physical shield for embryo protection but also as a long-term storage for active molecules such as hydrolytic enzymes and other substances that are released to the 'seedsphere' upon hydration.
Thus, in contrast to the well accepted idea that during programmed cell death macromolecules such as proteins are degraded and their constituents remobilized to other parts of the plants, we show that the essentially dead organs enclosing embryos, namely dead seed coat and possible remnants of endosperm store and release upon hydration various proteins including active hydrolases (nucleases, proteases, chitinases) as well as other substances that could inhibit microbial growth and might have the potential of enhancing seed persistence in the soil as well as germination and seedling establishment.
We hope you find our manuscript suitable for PLOS One.

Sincerely,

Gideon Grafi

Raviv et al Manuscript
Click here to download Manuscript Raviv et al Manuscript.docx

Dead Organs Enclosing Embryos Function as Long-Term Storage for Active Hydrolytic Enzymes

Short title:

Long-term storage of active proteins in dead organs enclosing embryos

Buzi Raviv[1], Lusine Aghajanyan [1], Gila Granot [1], Vardit Makover [2], Omer Frenkel [3], Yitzchak Gutterman [1] and Gideon Grafi [1]*

[1]French Associates Institute of Agriculture and Biotechnology of Drylands and [2]The Zuckerberg Institute for Water Research, The Institutes for Desert Research, Ben-Gurion University of the Negev, Midreshet Ben-Gurion, 84990, Israel; [3]Department of Plant Pathology and Weed Research, ARO, The Volcani Center, Bet Dagan 50250, Israel

*Corresponding author:

Email: ggrafi@bgu.ac.il (GG)

Abstract

Seed development culminates in programmed cell death (PCD) and hardening of organs enclosing the embryo (e.g., pericarp, seed coat, endosperm) providing essentially a physical shield for protection during storage in the soil. It is commonly believed that during PCD macromolecules such as proteins are degraded and their constituents remobilized into other plant parts. We examined the proposal that dead organs enclosing embryos are unique entities that store and release upon hydration active proteins that might increase seed persistence in soil, germination and seedling establishment. Proteome analyses of dead seed coats of Brassicaceae species revealed hundreds of proteins being stored and released upon hydration many are stress-associated proteins such as nucleases, proteases and chitinases. Functional analysis of exalbuminous seeds (endosperm is consumed and undergoes death during maturation) of Brassicaceae and cotylespermous seeds (endosperm is short lived and cotyledons assume the function of storage tissue) of Fabaceae species revealed that dead seed coats and possibly remnants of the endosperm tissue function as long-term storage for multiple active hydrolytic enzymes (e.g., nucleases) that can persist in active forms for decades. Substances released from the dead seed coat of the annual desert plant *Anastatica hierochuntica* displayed strong antimicrobial activity. Our data highlighted a previously unrecognized feature of dead organs enclosing embryos (e.g., seed coat, endosperm) functioning not only as a physical shield for embryo protection but also as a long-term storage for active proteins and other substances that are released upon hydration to the “seedsphere” and could contribute to seed persistence in the soil, germination and seedling establishment.

Introduction

The seed coat is a major defense against harmful environmental conditions protecting the embryo from mechanical stress as well as from microorganism invasion and from temperature and humidity fluctuations during storage. The seed coat is of maternal origin and is derived from the integuments (inner and outer) surrounding the ovule. However, it is not clear whether the dead seed coat was evolved to provide just a passive, physical shield for embryo protection or may represent also an active entity that stores and releases upon hydration substances (proteins, metabolites) that aid in seed persistence and longevity, germination and seedling establishment.

During *Arabidopsis* seed development five cell layers can be distinguished in the seed coat. The outer two cell layers having noticeable vacuoles derived from the outer integument, while three cell layers are derived from the inner integument [1]; the middle cell layer of the inner integument only surrounds part of the embryo sac [2]. The innermost cell layer of the inner integument, also known as endothelium, becomes vacuolated soon after fertilization and accumulates pigments mostly pro-anthocyanidins (PAs) [3]. Mucilage starts to accumulate during the torpedo stage in the outer cell layer of the outer integument. In *A. thaliana*, castor bean and tomato seeds the endosperm is undergoing programmed cell death (PCD) commonly prior to occurrence of PCD in the integuments [4-6]. At maturity, the endosperm, which is consumed during development in many Brassicaceae and Fabaceae species is dead [7]. Also, all cell layers of the seed coat are dead; most cell layers are crushed together except for the epidermis, that is, the outer cell layer of the outer integument [8,9]. In *Arabidopsis*, as well as in other Brassicaceae species, the outer cell layer of the seed coat functions as a special type of secretory cells that synthesize large amounts of pectinaceous mucilage during seed development and maturation [10,11]. This mucilage is rapidly swelled during hydration generating a gelatinous capsule around

the seed that functions in seed dispersal and adhesion to soil as well as providing a water reservoir for increasing success of germination, particularly under water scarcity [12-14]. In addition, seed coat mucilage may be important for maintaining the DNA repair mechanism within the embryo and thus assists in seed viability and consequently in maintaining a functional seed bank in hostile environments [15,16]. There are reported data implicating mucilage as a barrier regulating diffusion of water and oxygen to the inner tissues to prevent germination under inappropriate conditions [14]. Notably, *Arabidopsis* mucilage mutants, including *ttg1, gl2, atsbt1.7* and *dcr-1* germinated normally under non-stressed conditions but displayed reduced germination and seedling establishment in the presence of polyethylene glycol [17-19].

Perhaps, seed germination represents the most vulnerable stage of plant development as seeds are germinated into a potentially hostile, stressful environment. Furthermore, during storage in the soil, seeds are often subjected to vulnerable biotic and abiotic conditions (microbe attack, humidity and temperature fluctuations), which could affect longevity and persistence of seeds. Yet, seeds of many plant species persist in the soil and maintain viability for many years [20]. The mechanisms underlying seed persistence and viability in the soil have been addressed mostly with respect to chemical defense (secondary metabolites). For example, examination of seeds of over 80 plant species from the British flora and of some agricultural important weeds revealed that seeds contain at least traces of hydroxyphenols and many of them released hydrogen cyanide upon hydration, which might provide a defense layer against microbes and seed herbivores [21]. Seeds of various plant species such as *Raphanus sativus*, *Vigna unguiculata* (cowpea), *Phytolacca americana and Mirabilis jalapa* were found to contain and secrete various proteins including β-1,3-glucanases and small, anti-fungal proteins (AFPs) with strong activity against fungal pathogens and Gram-positive bacteria [22-25]. Yet, it is not clear if the origin of

these substances is maternal (e.g., seed coat) or zygotic (i.e., embryo). Notably, pigments in the maternally-derived seed coat that resulted from production of phenolic compounds (e.g., tannins) are often associated with antioxidant content and defense activity against pathogens [26-28]. It should be mentioned that proteome analysis of live seed coats was performed for developing seeds of soybean (*Glycine max* L.) revealing multiple proteins including an abundant protein of about 32 kDa class I chitinase [29] that plays a role in plant defense against pathogens [30-31].

Presently, no data exist on the role of dead organs enclosing embryos namely, seed coat and endosperm in storing of proteins, their composition and their endurance in active forms. We hypothesized that mature seeds and particularly the maternally-derived seed coat and probably other hardened parts of plant dispersal units (Raviv et al., manuscript submitted) store proteins and other substances (e.g., antimicrobial substances, nutrients) that are released to the seed's immediate surroundings ('seedsphere') upon hydration and assist in seed persistence and longevity, germination and seedling establishment. Indeed, analysis of proteins released from seeds of *Arabidopsis thaliana* and from seed coats of *Sinapis alba* and *Anastatica hierochuntica* revealed multiple proteins being released upon hydration including proteins involved in stress response, many function as hydrolases such as nucleases, proteases and chitinases. In gel assays demonstrated that hydrolases can persist for decades in active forms within the dead seed coats. Substances released from embryos or seed coats of the annual desert plant *Anastatica hierochuntica* have strong antimicrobial activities. Thus, our data highlighted the potential function of dead organs enclosing embryos, that is the seed coat and remnants of the endosperm, in storing and releasing upon hydration multiple substances that might explain, at least partly, how seeds persist and retain viability in the soil for many years.

Materials and methods

Plant materials, secretion of substances and mucilage staining

Seeds of various wild crucifers and leguminous species were collected in the field in Israel or obtained from the Israel Plant Gene Bank (Supplementary Table S1). Seeds of *Arabidopsis thaliana* (Col and Ler) wild type and mucilage mutant plants *gl2* and *mum4* (kindly provided by T. Western and S. Harpaz-Saad) were collected from plants grown in growth room at 22°C±2°C under long day photoperiod. Ten mg of Arabidopsis seeds or seeds equivalent to 10 mg Arabidopsis seed surface area, or dissected seeds (embryos and seed coats) were incubated in 100 μl of phosphate-buffered saline (PBS) at 4°C for 8-12 h after which the aqueous phase was collected, centrifuged (4°C, 11,000 rpm, 5 min) and the supernatant was used immediately or stored at -20°C until used.

Mucilage was stained with Ruthenium red essentially as described [32]. Seeds were hydrated with either distilled water or 50 mM EDTA for 90 min, washed with distilled water and incubated with 0.01% Ruthenium Red (Sigma-Aldrich) 90 min. The seeds were washed with distilled water to remove access dye and observed under a binocular microscope (Zeiss).

Proteome analysis

Proteome analysis of seeds and seed coats were performed by the proteomic services of The Smoler Protein Research Center at the Technion, Israel. Proteins released from *Arabidopsis* seeds or seed coats of *Sinapis alba* and *Anastatica hierochuntica* following hydration (4°C, 12 h in PBS) were digested with trypsin followed by separation and mass measurement on LC-MS/MS

on LTQ-Orbitrap and identification by Discoverer software software against the uniprot database, which contains plants and fungi proteins and against a decoy database in order to determine the false discovery rate. All the identified peptides were filtered with high confidence, top rank, mass accuracy, and a minimum of 2 peptides. High confidence peptides were passed the 1% FDR threshold (FDR =false discovery rate, is the estimated fraction of false positives in a list of peptides). At least 2 replicates were performed for each examined organs. Semi-quantitation was done by calculating the peak area of each peptide. The area of the protein was calculated from the average of the two to three most intense peptides from each protein.

We applied a more stringent filtering to the proteins, which are considered present in each plant. Considering that the average amino acid length of the proteins in the dataset was nearly 400 and assuming that 2 peptides are the minimum requirement for an average protein, we extrapolated this cutoff to consider protein length as the following: The minimum number of peptides per protein is the protein length divided by 200, but not less than 2 peptides. Additionally, the protein coverage by peptides from all samples must be higher than 10% for the Arabidopsis seed secretion proteome data (Supplemental data set 1) and 20% for the analysis of seed coats of *Sinapis alba* and *Anastatica hierochuntica* (Supplemental data set 2). Therefore, a protein is regarded as “present” in a sample if the number of peptides is above the threshold for that protein length, the signal is >0, and the total peptide coverage is either >10% or >20%. A protein is regarded as “present” in a plant if it is “present” in at least two replicates of that plant.

GO categorization analysis was carried out using the BiNGO (v3.0.3) plugin of Cytoscape (v3.4.0). GO ontology file (go.obo) was downloaded from geneontology.org (http://purl.obolibrary.org/obo/go.obo). Annotation of UniProt accessions with GO terms was according to UniProt. For the reference set we used the 246 proteins, which were present in at

least one of the plants. No statistical test for GO enrichment was carried out, and only the number of proteins in each GO term was counted. The following GO trees were examined, Biological Process (BP), Molecular Function (MF) and Cellular Component (CC). GO Slim for plants, which included broad categories of BP, MF and CC was also performed and the results were sorted by descending number of proteins per GO term.

In-gel nuclease assay

Nuclease assay was performed essentially as described [33] in polyacrylamide gel containing 300 μg/ml denatured salmon sperm DNA or ribonucleic acid from Torula yeast (Sigma) for RNases activity. Briefy, proteins released from 2 mg seeds, embryos or seed coats were incubated with sample buffer containing 2% SDS, 62.5mM Tris pH 6.8 and 10% glycerol and bromophenol blue for 1h at 37°C followed by separation on SDS/PAGE (samples were not boiled). The gel was washed twice, each time for 30 min, at room temp in buffer containing 10 mM Tris-HCl pH 7.5 and 25% isopropanol, followed by washing twice 15 min each with 10 mM Tris-HCl pH 7.5. Nuclease activity was performed by incubating the gel with 10 mM Tris-HCl pH 7.5 with or without divalent cations. We used either combination of 10 mM $MgSO_4$ and 10 mM $CaCl_2$ or only 10 mM $ZnCl_2$ as cofactors for 75 min at 37°C. The gel was stained for 60-80 minutes with 10 mM Tris HCl pH 7.5 containing 2 μg/ml ethidium bromide and inspected under UV light.

Endonuclease conversion assay

S1-type endonuclease activity was assessed by the conversion assay, that is the capability to convert supercoiled plasmid DNA into relaxed and linear forms essentially as described [34]. Briefly, 1 μg of supercoiled plasmid DNA was incubated with seed secretion in buffer containing

10 mM Tris-HCl pH 7.5, 10 mM $MgSO_4$ and 10 mM $CaCl_2$. Samples were incubated at room temperature for various time periods and reactions were stopped by adding EDTA to a concentration of 50 mM. The conversion of supercoiled plasmid DNA into relaxed and linear forms was monitored after separation on 1% agarose gel containing ethidium bromide.

In-gel chitinase assay

In gel chitinase assay was performed essentially as described [35]. Briefly, Intact seeds of *Raphanus sativus*, seed coats and isolated embryos were incubated in 0.1M NaHPO4 (pH 6) at 4°C for 16 h, after which the sup was collected and secretion from 2 seeds equivalent were taken for separation on SDS/PAGE. Samples were first incubated in chitin sample buffer (15% sucrose, 2.5% SDS, 12.5 mM Tris-HCl pH 6.7, 0.01% Bromophenol Blue) for 1 h at 37°C and samples were run on 12% SDS/PAGE containing 0.01% glycol chitin. The gel was incubated in buffer containing 100 mM sodium acetate (pH=5.2) and 1% triton x-100 for 2 h at 37°C followed by staining for 5 min with 0.01% calcofluor white in 500 mM Tris-HCl (pH=8.9). The gel was washed with distilled water for 1 h and visualized by UV transillumination.

Antimicrobial assays

The spectrophotometric bioassay for microbial sensitivity was performed essentially as described [36]. Briefly, we used *Staphylococcus aureus* as a representative model for Gram-positive bacteria. One colony of each strain was suspended in 10 ml of LB Broth (Difco, MD, USA) and grown overnight at 37°C. The cultures were diluted, transferred to 25% LB Broth and grew at 37°C to 0.03-0.05 optical density (OD_{595}; Epoch, Biotek, USA). A 150 μl aliquot of the culture was incubated with 50 μl of PBS, ampicillin (final concentration 100 μg/ml) or with 50 μl of

substances released from whole seed, embryo, or the seed coat (3-9 replicates per treatment) in a flat-bottom 96-well microtiter plate. Plates were incubated in the dark using a spectrophotometer (Synergy 4, Biotek, USA) and reads (OD_{595}) were taken at intervals of 30 minutes in a course of 24 hours. The average OD for each blank replicate at a given time point was subtracted from the OD of each replicate treatment at the corresponding time point and standard errors were calculated for each treatment at every time point.

Fungi spore germination assay

Microconidia of the pathogenic fungi *Fusarium oxysporum* f.sp. *melonis* as a model pathogen were obtained from two-week-old mycelium cultivated on potato dextrose agar plates and diluted in double distilled water to a level of 10^4 microconidia per ml. Substances released from seeds (in PBS) were added to potato dextrose broth in a 1:1 ratio, and 63 µl of the mixture was vortexed with 7 µl of spore suspension and co-cultivated on a depression slide in a dark, moist chamber at 25ºC. Conidial germination was monitored after 24 h under light microscope (Zeiss, Germany).

Results

Seeds release multiple proteins upon hydration – proteome analysis

To gain insight into proteins released from seeds upon hydration and might contribute to seed germination and seedling establishment, we initially incubated seeds of *Arabidopsis thaliana* (Col) in PBS buffer at 4°C for 12 h and the supernatant was collected and subjected to proteome analysis using LC-MS/MS followed by identification by Discoverer software against the Uniprot database, which contains plants and fungi proteins; notably, only plant proteins were identified (Supplemental data set 1). Implementing the cutoffs (at least 2 peptides and 10% coverage) we identified 238 proteins that were released from Arabidopsis seeds following hydration (Supplemental data set 1). Functional classification using PANTHER classification system (Mi *et al.*, 2005) revealed a high proportion of proteins with catalytic activity (55.2%), while among protein classes, hydrolases (18.5%), oxireductases (17.3%) and nucleic acid binding proteins (17.9%) were prominent. Classification for biological processes showed that 41.2% of the released proteins are involved in metabolic processes and 5.7 % of the proteins are related to plant response to stimulus (Supplemental Fig. S1). Proteins responsive to stimulus include several plant defensin-like (DEFL) molecules also known as low molecular weight cysteine-rich (LCR) proteins encoded by At1g75830/AtPDF1.1, At2g12475/DEFL112 and At1g13607/DEFL286, as well as LCR17 (At4g11760) and LCR25 (At4g29305), which are implicated in defense response to fungus [37-39]. Other stress-related proteins include the pathogenesis related protein 5 (PR5, AT1g75040) and a thaumatin-like protein involved in response to pathogens [40]. Among hydrolases we identified multiple proteases and nucleases including several endopeptidases such as aspartyl proteases (At1g03220 and At3g54400), cysteine proteinase (At1g06260) and subtilisin-like serine endopeptidase (At5g03620),

endonuclease BFN1/ENDO1 (At1g11190) whose expression was studied with respect to senescence and cell death [41] and THIOGLUCOSIDE GLUCOHYDROLASE 1 (AtTGG1, At5g26000), an enzyme that catalyzes the hydrolysis of glucosinolates into compounds that are toxic to various microbes and herbivores [42].

Notably, identification of proteins was performed by the Discoverer software against the Uniprot database, which contains plants and fungi proteins, confirming that proteins released from *Arabidopsis* seeds upon hydration (at 4°C) are of plant origin and not derived from microbes. In addition, no bacterial growth is apparent when non-sterile seeds of *Arabidopsis thaliana*, *Diplotaxis erucoides* and *Sinapis alba* were incubated on MS plate at 4°C for 72 hours.

Proteins released from seeds following hydration are enzymatically active

The possibility existed that some of the hydrolases recovered in the proteome analysis are partially degraded and are not enzymatically functional. One enzyme released upon hydration is BFN1/ENDO1 endonuclease implicated in senescence and PCD. We analyzed for endonuclease activity by in gel nuclease assays using denatured salmon sperm DNA as substrate and various cations as cofactors. Here we extended the analysis to measure nuclease activity in substances released from various mucilaginous seeds of Brassicaceae species. To this end, we collected in the field seeds from wild crucifers including *Capsela bursa-pastoris* (L.) Medik., *Sisymbrium irio* L., *Moricandia nitens* (Viv.) E. A. Durand & Barratte, *Diplotaxis erucoides* (L.) DC., *Diplotaxis harra* (Forssk.) Boiss. and *Sinapis alba* L. Results showed (Fig. 1) that all examined seeds of wild species released nucleases upon hydration similarly to *Arabidopsis*. In all cases, no or very

low activity was observed in the absence of cations or in the presence of Zn^{2+} but high nuclease activities at positions of about 22 and 35 kDa were recovered in the presence of Ca^{2+} and Mg^{2+}.

Fig. 1. Nuclease activities in seed secretions of Brassicaceae species. In-gel nuclease assay demonstrating high nuclease activity in secretions from seeds of various crucifers. Seeds of *Arabidopsis thaliana* Ler and Col lines, *Capsella bursa-pastoris*, *Sysimbrium irio*, *Moricandia nitens*, *Diplotaxis erucoides*, *Diplotaxis harra* and *Sinapis alba* were incubated in PBS for 8 h at 4°C, the aqueous phase was collected and proteins were separated on 12% SDS/PAGE containing denatured salmon sperm DNA. Nuclease reaction was performed with or without the indicated divalent cations and activity was visualized by staining with ethidium bromide. M, protein size markers. Note that strong nuclease activity was recovered in the presence of $Mg^{2+/}Ca^{2+}$.

Most nucleases are released from the dead organs enclosing the embryo

Nucleases released from the dry seed upon hydration could have been released from the embryo or from dead organs enclosing the embryo, namely, the seed coat and possibly also remnants of the endosperm (will be referred to as seed coat). To test the origin from which nucleases are released, we separated seed coat from the embryo of three crucifer species having relatively large seeds, namely, *S. alba*, *D. harra* and *Anastatica hierochuntica* L. Separated seed coats and embryos were hydrated, the sup was collected and analyzed for nuclease activities using in gel nuclease assay. Results showed (Fig. 2A) that nucleases were released from the seed coat of all examined species but not from the embryo itself. We also analyzed for the presence of ribonucleases in substances released from *S. alba* seed using in-gel assays with yeast Torula RNA as a substrate. Results showed (Fig. 2B) the presence of several differentially migrating ribonucleases ranging from 27 to 37 kDa in substances released from both the embryos and seed coats; high RNase activities were recovered from the seed coat of *S. alba*.

Fig. 2. Nucleases are released from the seed coat. A, Proteins released from seeds, seed coats and embryos of the indicated Brassicaceae species were subjected to in gel nuclease assay using denatured salmon sperm DNA as substrate. Upper panels are the EZBlue staining and the lower panels are the nuclease assays. M, protein molecular weight markers. B, In gel RNase assay. Proteins released from seeds, seed coats and embryos of *S. alba* were subjected to in gel nuclease assay using Torula yeast RNA as substrate. Asterisks at the lower panel mark positions of active RNases. Upper panel is the EZBlue staining gel. C, In vitro endonuclease assay. Supercoiled plasmid DNA (1 μg) was incubated for the indicated time points at room temperature with *S. alba* seed secretion. Supercoiled plasmid DNA incubated with the buffer only or with 50 mM EDTA only, for 15 min, were included as controls. Lin is restriction enzyme linearized plasmid (marked by white asterisk). The positions of the different topological forms of plasmid DNA are indicated: R, relaxed form; L, linear; SC, supercoiled plasmid DNA. M, molecular size markers of 1 kb DNA ladder.

The timing by which nucleases are released from seeds upon hydration was relatively very fast and occurs within 15 min following hydration; maximal nuclease activity was observed after 30 min of hydration (Supplemental Fig. S2A). We also analyzed the dynamics of release of nucleases from seed coats of *S. alba* by repeated extraction with PBS. The Results showed that most nucleases were released in the first extraction round and then gradually decreased (Supplemental Fig. S2B).

Although Brassicaceae seeds are exalbuminous, that is, the endosperm is consumed during embryo development and undergoes cell death its remnants are often firmly associated with the dead seed coat. To ease with separation of the seed coat from remnants of the endosperm, we also analyzed seed coats of plants having cotylespermous seed storage tissue type such as legumes, in which the cotyledons assume the function of storage tissue. In most legumes, the endosperm is short-lived and it is absorbed during seed development and at maturity it is not present or is visible as a thin layer surrounding the cotyledons or the embryo [43,44]. We have

selected seeds of three leguminous species, namely, *Lupinus pilosus* L., *Cicer arietinum* L. (cultivated chickpea) and *Colutea istria* Miller and the seed coats were separated from embryos and analyzed for nucleases released upon hydration. Results showed that all examined leguminous species store and release nucleases exclusively from the seed coat (Supplemental Fig. S3), further supporting the notion that the essentially dead seed coats enclosing the embryo in a variety of plant species store and release active nucleases upon hydration. To show that storage within the dead seed coat is a general phenomenon in *planta*, we extended our study to three leguminous species, namely, *Lupinus pilosus* L., *Cicer arietinum* L. (cultivated chickpea) and *Colutea istria* Miller. The seed coats were separated from embryos and analyzed for activity of nucleases released upon hydration. Results showed that all examined leguminous species store and release nucleases exclusively from the seed coat (Supplemental Fig. S3), further supporting the idea that storage of hydrolases within the dead seed coat is a general theme in plants.

S1-type endonucleases are released from the seed coat

The 35 kDa nuclease released upon hydration from seeds of *Arabidopsis* and other examined species is the predicted molecular mass of BFN1/ENDO1 (identified in the proteome data), a nuclease related to S1-type endonucleases. S1-type endonucleases, such as *Aspegillus* S1 nuclease and mung bean nuclease are widely used in molecular biology applications and are capable of introducing nicks and double strand DNA breaks (DSBs) into supercoiled plasmid DNA converting it to relaxed and/or linear forms - a well-established method for monitoring single-strand DNA endonucleases [45]. The capacity of single-stranded DNA endonucleases to target and change the topology of double-stranded supercoiled plasmid DNA is a consequence of torsional strain generated in superhelical DNA, which promotes local denaturation and unpairing

essentially at weakly hydrogen-bonded regions [46]. To monitor for specific S1-type endonuclease activity we incubated supercoiled plasmid DNA with proteins released from *S. alba* seeds for various time periods (0.5 to 15 min) in the presence of Ca^{2+}/Mg^{2+} cations, reactions were stopped by adding chelating agent EDTA to 50 mM and samples were separated on 1% agarose gel containing ethidium bromide. Results showed (Fig. 2C) that substances released from *S. alba* upon hydration possess S1-type endonuclease activity that gradually converted supercoiled plasmid DNA into a linear form in two distinguishable steps. The first is seen by the gradual accumulation of the relaxed form concomitantly with gradual disappearance of the supercoiled plasmid DNA, which is followed by a second step of accumulation of the linear form. Thus, substances released from seeds contain S1 type endonucleases capable of introducing double strand DNA breaks into superhelical DNA.

Long-term persistence of active nucleases in dead seed coats

To test the endurance of nucleases within dead seed coats, we examined seeds of *A. hierochuntica* obtained from dried skeletons collected from the islands of Tiran and Sanafir at 1968 and stored at room temperature. Notably, when fruits matured, the *Anastatica* desiccated skeleton is rolled inward forming a ball shape structure that protect fruits and seeds from desiccation and predation, generating an aerial seed bank that remain viable for many years [47,48]. We first compared the germination capacity of 1968 seeds of Tiran and Sanafir ecotypes with those of 2015 seeds of Ovda and Sodom ecotypes. The results showed (Fig. 3) no germination of the 1968 Tiran and Sanafir seeds compared to almost full germination of *A. hierochuntica* seeds collected during the year of 2015 (Fig. 3A). Yet, both the 2015 and the 1968

seeds released upon hydration active nucleases (Fig. 3B) suggesting that nucleases can be well preserved for decades in dead organs enclosing the embryos.

Fig. 3. Long-term survival of nucleases within the dead seeds of *A. hierochuntica*. A, Germination test of new (2015) and old (1968) seeds of *A. hierochuntica* collected from various sites. Tiran and Sanafir ecotypes refer to islands located at the straits of Tiran that separate the Red Sea from the Golf of Aqaba/Eilat. Sodom ecotype was collected near Sodom mountain and the dead sea, while Ovda ecotype was collected near Eilat. B, Dead, non viable seeds of Tiran and Sanafir store and release upon hydration active nucleases. Seeds of the indicated ecotypes were incubated in PBS for 8 h, the aqueous phase was collected and analyzed by in-gel nuclease assay using denatured salmon sperm DNA as substrate. M is the protein molecular weight markers. Long-term survival of nucleases (C) and chitinases (D) within the seed coat of *Raphanus sativus* collected at 1980. The gels in (C) and (D) were stained with EZblue following the nuclease and chitinase reactions. Asterisks in (D) indicate chitinases released from the seed coat. M, Protein molecular weight markers.

Further analysis of seeds of *Raphanus sativus* collected at 1980 (provided by Israel Plant Gene Bank) showed that all hydrolase activities, namely nucleases (Fig. 3C) and chitinases (Fig. 3D) are released from the dead seed coat providing further support that nuclease as well as chitinase activities persist within the dead organs for decades.

Mucilage is not required for storage and release of hydrolases

Although many of the Brassicaceae species contain mucilage, *Raphanus sativus* as well as Fabaceae species are not suggesting that the mucilage is not an obligate requirement for storage and release of proteins. We wanted to test the importance of mucilage, in species naturally containing mucilage such as *Arabidopsis thaliana*, for storage and release of nucleases from the seed coat. To this end, we used several *Arabidopsis* mucilage mutants carrying mutation in genes whose products involved in the regulation of mucilage production, including GL2 and MUM4

[49]. Staining of seeds of Col and of the mucilage mutant *gl2* with ruthenium red demonstrated (as expected) the lack of mucilage in *gl2* (Fig. 4A). However, in gel nuclease assay demonstrated (Fig. 4B) that all mutant seeds released nucleases similarly to Col seeds.

Fig. 4. The seed coat mucilage is not required for storage and secretion of nucleases. A, Ruthenium red staining of *Arabidopsis* Col seeds and *gl2* mucilage mutant. B, In gel nuclease assay for proteins released from seeds of the indicated wild type and mucilage mutant lines. M, protein molecular weight markers.

The dead seed coats of *S. alba* and *A. hierochuntica* store and release multiple proteins involved in stress response: proteome analyses

It appears that seed coats of various species function as a major storage compartment for hydrolases such as nucleases and chitinases. We sought to identify proteins specifically released from the seed coat following hydration while attempting to address two aspects, namely, differences between *A. hierochuntica* ecotypes new and old collections and differences between different genotypes *A. hierochuntica* versus *S. alba*. To this end, we compared the proteome profiles of dead seed coats of *A. hierochuntica* (Sodom ecotype 2015) and *A. hierochuntica* (Tiran Island 1968) and that of *S. alba* (2015). Thus, proteins released from seed coats of *A. hierochuntica* (2 replicates each ecotype) and *S. alba* (3 replicates) were subjected to proteome analysis using LC-MS/MS followed by identification by Discoverer software against the Uniprot database, which contains plants and fungi proteins; only plant proteins were identified (Supplemental data set 2). Implementing the cutoffs described in Material and methods we identified 246 proteins that are present in at least one plant (Supplemental data set 3). Accordingly, 71 proteins were identified in Sodom ecotype (2015), 77 in Tiran Island ecotype

(1968) and 145 proteins were identified in seed coat of *S. alba* (Fig. 5A). Venn diagram also shows that 45 proteins are shared by all species/ecotypes examined and 50 proteins out of 98 are shared by *Anastatica* ecotypes Sodom (2015) and Tiran (1968). Notably, out of 98 proteins identified in both *Anastatica* ecotypes, 77 proteins were also released from *S. alba* seed coat (Supplemental data set 3). Functional categorization of these 77 proteins revealed that among the 47 proteins recognized in biological process category, 40 proteins are involved in metabolic processes, 20 in oxidation-reduction processes and 15 proteins are related to response to stress (Fig. 5B). Molecular function analysis (Fig. 5C) revealed that among the 64 proteins recognized in this category, 42 proteins have catalytic activity including lyase activity (9 proteins), oxireductase activity (8 proteins) and hydrolases (8 proteins including chitinases, peptidases and lipases).

Fig. 5. Analysis of proteins released from the dead seed coats of *A. hierochuntica* and *S. alba*. (A) Venn diagram showing the number of proteins recovered from the dead seed coats of each species examined and the number of proteins shared between the indicated species. 77 proteins shared by *Anastatica* ecotypes and *S. alba* highlighted yellow. (B and C) GO categorization for biological process and molecular function, respectively, of the 77 proteins shared by all species examined.

Analysis of microbial growth controlling activity

A. hierochuntica is commonly used in traditional medicine [50] and the methanolic extract of the plant was reported to contain antimicrobial activity [51]. These prompted us to investigate whether *Anastatica* seeds also store antimicrobial substances that are released upon hydration and what is the origin of these substances, the embryo or the seed coat. We used the Gram-positive strain *Staphylococcus aureus* for antibacterial study. *S. aureus* was grown in a flat-bottom 96-well microtiter plate in LB medium supplemented with PBS, ampicillin or with substances released from seed coats or embryos of *A. hierochuntica*. Plates were incubated in the dark using

a spectrophotometer and reads (OD_{595}) were taken at 30 min intervals. Results showed (Fig. 6A) that strong antibacterial activity is released from both *A. hierochuntica* embryos (*Ah* embryo) and seed coats (*Ah* coat), which was comparable to the inhibitory effect of ampicillin (100 μg/ml). We also found that substances released from intact seeds of *A. hierochuntica* inhibited conidial germination of the pathogenic fungus *Fusarium oxysporum* f.sp. *melonis* (Fig. 6B). Notably, seeds collected at 1968 from the islands of Tiran and Sanafir, similarly released substances with a strong antibacterial activity.

Fig. 6. Seeds of the desert plant *Anastatica hierochuntica* release antimicrobial substances. A, *Staphylococcus aureus* was grown in a flat-bottom 96-well microtiter plate in the presence of PBS, ampicillin (100 mg/L) or in the presence of substances released from embryos (*Ah embryo*) or seed coats (*Ah coat*) of *A. hierochuntica*. Bacterial growth was monitored by measuring the OD_{595} of the culture at 30 min. intervals in the course of 24 h. Each treatment was performed in triplicates and error bars represent the standard deviation. B, *A. hierochuntica* seeds released substances that inhibit conidiospore germination. Microconidia derived from *Fusarium oxysporum* f.sp. *melonis* were mixed with potato dextrose broth (PDB) alone (control) or with PDB supplemented with *A. hierochuntica* seed released substances. Mixtures were placed on depression slides and incubated in moist chamber for 24 h at 25°C in a dark and inspected under a light microscope. mc, microconidia.

Discussion

The seed coat and other hardened parts enclosing the embryo are commonly considered as a passive barrier protecting the embryo from harmful environmental conditions. Here we showed that the dead organ enclosing embryos functions as a storage for active hydrolases and antimicrobial substances that might play important role in regulating seed longevity, germination and seedling establishment. Indeed, the seed coat has been implicated in dormancy and seed longevity and quality [52] and some reports have also suggested the function of the seed coat in storing antimicrobial substances such as tannins, flavonoids and anti fungal proteins that may protect the embryo and germinating seeds from pathogens [27,53].

In the present work, proteome analyses of dead seed coat of *S. alba and A. hierochuntica* revealed multiple proteins being released upon hydration. Many of these proteins are related to stress response and host defense and include hydrolases (chitinases, proteases and nucleases) and antifungal proteins. Interestingly, the proteome data revealed that the two *Anastatica* ecotypes are clustered together sharing 50 proteins out of the 98 identified in both ecotypes. Importantly, the long-term storage within the dead seed coat of *Anastatica* Tiran ecotype (collected at 1968) not only did not affect mass measurement and protein identification but also had no significant effect on enzymatic activities as demonstrated by in gel nuclease assays. Furthermore, it appears that storage of proteins within the dead seed coat is a general phenomenon in plant seed biology; 78% of the proteins found in *Anastatica* ecotypes (77 proteins) were also found in *S. alba* seed coat. Functional categorization of these proteins revealed overrepresentation of proteins related to stress response including three cysteine-rich antifungal proteins related to *Arabidopsis* PDF1.1/LCR67 (At1G75830) and PDF1.2b (AT2G26020). Cystein-rich antifungal proteins are small proteins known as plant defensins [22] that are present in all plant families and can confer enhanced resistance to pathogen when overexpressed in transgenic plants [22,54]. These proteins were primarily found in seeds but are also present in leaves and flowers and often upregulated following pathogenic attack or in response to environmental stress such as drought [55]. Notably, the proteome data revealed xyloglucan endotransglucosylase/hydrolase (XTH), a xyloglucan modifying enzyme, which is thought to play a key role in fruit ripening by loosening the cell wall [56]. This enzyme may assist in loosening of cell walls of the testa and endosperm to allow their rupture and consequently radicle protrusion. This is supported by the findings that in tomato, expression of the XTH gene, SlXET4 was induced by gibberellic acid (GA) in the micropylar endosperm (a region restricting radicle protrusion) during germination [57]. Also, in *Lepidium*

sativum the analysis of putative cell-wall-loosening genes (expansins and XTHs) showed that their transcripts are accumulated to high level in the micropylar endosperm 8 h after imbibition [58]. However, in contrast with tomato germinating seeds, in *L. sativum* seeds, GA significantly reduced abundance of XTH18 and XTH19 in the micropylar endosperm [58]. Other proteins identified include heat shock proteins HSP70T-1 (related to At1g51090), which is involved in plant immunity and HSP70B (related to At1g16030), peroxidase 12 (related to Arabidopsis At1G71695), as well as chitinases and endochitinases involved in response to pathogens [59,60]. We also identified storage proteins cruciferins that suggest that the seed coat may also contain remnants of the dead endosperm tissue.

Focusing on nucleases, we showed that dead seed coats possess highly active DNases and RNases that can persist in active forms for decades. Some of the DNases appeared to be single stranded DNA endonucleases that are belong to the S1-type endonucleases. This was confirmed by their capacity to convert supercoiled plasmid DNA into relaxed and linear forms, a characteristic feature of S1-type endonucleases such as *Aspergillus* S1 and mung bean nucleases [34,40]. The significance of these nucleases for seed germination and seedling establishment is yet unknown. Endonucleases in general have been implicated in diverse cellular processes including DNA synthesis and DNA repair [61] (Balakrishnan and Bambara, 2013) as well as in fragmentation of genomic DNA during PCD [62-64]. Endonucleases associated with programmed cell death both in plants and animals require Ca^{++} and Mg^{++} for activity [65,66], which is consistent with the requirement of these cations for activity of endonucleases released from seed coats of various crucifers. The capacity of endonucleases to target unpaired regions within superhelical DNA to introduce nicks and DSBs may implicate them as seed defense factors against plasmid-containing soil pathogens. For example, the *Clavibacter michiganensis*

subsp. michiganensis, a Solanaceae species-pathogenic Gram-positive actinomycete contains two plasmids, designated pCM1 and pCM2, which are important for its pathogenic activity, inasmuch as plasmid-free derivative CMM100 can colonize tomato, but showing no disease symptoms [67]. Targeting these superhelical plasmids by endonucleases released from seeds can lead to neutralization of virulent genes and conversion of a potentially pathogen into a non-pathogenic one. Also, RNases were shown to inhibit growth of pathogenic fungi. Accordingly, exogenous application of S-like RNase NE into the extracellular space of leaves inhibits the development of *Phytophthora parasitica* [68,69], a oomycete soilborne pathogen with a wide range of host plants. Also, the Wheatwin1 PR4 RNase was shown to enter inside fungal cells without affecting the integrity of cell walls and possesses antifungal activity, which is dependent on its enzymatic activity [70]. In-gel RNase assays showed the activity of multiple RNAses released from the seed coat and the embryo of various Brassicaceae species further highlighting the wide range of released molecules that have the potential to act as pathogen inhibitors. Notably, the proteome analysis revealed multiple proteins that could act against pathogens including chitinases and endochitinases. Chitinases are enzymes that degrade chitin an abundant polysaccharide found in a variety of organisms including insects, fungi, yeast, and algae. Chitinase and glucanase genes were often over-expressed in plants to confer resistance against fungal pathogens [71-73].

Proteome analysis was performed for the seed coats (testa) of soybean (Glycine max L. Merr. cv Jack) during various stages of seed development [74]. Interestingly, at S9 stage where seeds are quiescent, yellow/tan-colored, and fully dehydrated the authors have observed a relatively more proteins related to stress response (~15%). The proteome data of both *Anastatica* and *Sinapis* seed coats showed overrepresentation of proteins (15 out of 47; ~32%) involved in stress response (Fig. 5B), suggesting that these proteins are accumulated in the dead seed coats

and might provide an additional active defense layer for embryo protection.

Presently, we could not find a clear correlation between hydrolase activities and inhibition of bacterial growth directed by substances released from *A. hierochuntica.* Accordingly, both embryo and seed coats released strong antimicrobial activity, yet nucleases were released exclusively from the seed coat suggesting that nucleases may not be the principal inhibitory factor(s) of bacterial growth. Alternatively, a metabolite or combination of metabolites may provide the principal factor(s) inhibiting bacterial growth. Many such metabolites with antimicrobial activities exist in plants including alkaloids, phenolic compounds and terpenoids [75].

Conclusions

The finding that proteins are stored and remained active within essentially dead organs of the seed coat for many years is puzzling. It is commonly believed that cellular proteins undergo complete degradation when cell die and their constituents are remobilized to other parts of the plants (young leaves, fruits, embryos). The results presented here suggest that seed coat and also other hardened parts enclosing embryos (e.g., glumes, lemmas and palease in Poaceae species, Raviv et al. manuscript submitted) were evolved not just for providing a physical shield for embryo protection but also as storage organs for multiple active proteins and probably metabolites and other substances for the purpose of nourishment as well as protection of germinating seeds from soil pathogens, which may facilitate seed persistence in the soil, germination and seedling establishment in wild as well as in agroecosystem [76].

Considering the sessile nature of plants and that the seed coat is a maternally derived organ, our future goal is to explore how exposure of mother plants to biotic and abiotic stresses during

flowering and seed maturation affects the composition and activities (e.g., hydrolase activities, antimicrobial activities) of substances stored in and released from seed coats upon hydration as well as the effect of the seed coat on seed germination and seedling establishment. The realization that plant dead organs store live molecules and possibly multiple beneficial substances might change the way we treat and refer to plant remnants in agricultural practices as well as the way we store seeds in seed banks.

Acknowledgements

We thank T.L. Western and S. Harpaz-Saad for providing seeds of *Arabidopsis* mucilage mutants and the Israel Plant Gene Bank for providing seeds of wild species. We also thank S. Harpaz-Saad for helping with mucilage staining, Fauzi Abu-Moch and Mr. Lior Gur for technical support of the conidia germination experiments. We thank the Harbour Foundation for supporting B.R. research. The work was supported in part by the Margolin foundation to G. Grafi.

References

1. Beeckman T, De Rycke R, Viane R, Inze D. Histological study of seed coat development in Arabidopsis thaliana. J. Plant Res. 2000; 113: 139–148.
2. Schneitz K, Hulskamp M, Pruitt RE. Wild-type ovule development in Arabidopsis thaliana: a light microscope study of cleared wholemount tissue. Plant J. 1995; 7: 731–749.
3. Devic M, Guilleminot J, Debeaujon I, Bechtold N, Bensaude E, Koornneef M, Pelletier G, Delseny M. The BANYLUS gene encodes a DFR-like protein and is a marker of early seed coat development. Plant J. 1999; 19: 387–398.
4. Nakaune S, Yamada K, Kondo M, Kato T, Tabata S, Nishimura M, Hara-Nishimura I. A vacuolar processing enzyme, deltaVPE, is involved in seed coat formation at the early stage of seed development. Plant Cell 2005; 17: 876-887.
5. DeBono AG, Greenwood JS. Characterization of programmed cell death in the endosperm

cells of tomato seed: two distinct death programs. Can. J. Bot. 2006; 84: 791–804.

6. Schmid M, Simpson D, Gietl C. Programmed cell death in castor bean endosperm is associated with the accumulation and release of a cysteine endopeptidase from ricinosomes. Proc. Nat. Acad. Sci. USA. 1999; 96: 14159-14164.

7. López-Fernández MP, Maldonado S. Programmed cell death in seeds of angiosperms. J. Integr. Plant Biol. 2015; 57: 996-1002.

8. Windsor JB, Symonds VV, Mendenhall J, Lloyd AM. Arabidopsis seed coat development: morphological differentiation of the outer integument. Plant J. 2000; 22: 483-493.

9. Haughn G, Chaudhury A. Genetic analysis of seed coat development in Arabidopsis. Trends Plant Sci. 2005; 10: 472-477.

10. Willats WG, McCartney L, Mackie W, Knox JP. Pectin: cell biology and prospects for functional analysis. Plant Mol. Biol. 2001; 47: 9-27.

11. Caffall KH, Mohnen D. The structure, function, and biosynthesis of plant cell wall pectic polysaccharides. Carbohydr. Res. 2009; 344: 1879-1900.

12. Gutterman Y, Shem-Tov S. Mucilaginous seed coat structure of Carrichtera annua and Anastatica hierochuntica from the Negev Desert highlands of Israel and its adhesion to the soil crust. J. Arid Environ. 1997; 35: 695–705.

13. Sun Y, Tan DY, Baskin CC, Baskin JM. Role of mucilage in seed dispersal and germination of the annual ephemeral Alyssum minus (Brassicaceae). Aus. J. Bot. 2012; 60: 439-449.

14. Yang X, Baskin JM, Baskin, C.C, Huang, Z. More than just a coating: Ecological importance, taxonomic occurrence and phylogenetic relationships of seed coat mucilage. Perspect. Plant Ecol. Evol. Syst. 2012, 14, 434–442.

15. Huang Z, Boubriak I, Osborne DJ, Dong M, Gutterman Y. Possible role of pectin-containing mucilage and dew in repairing embryo DNA of seeds adapted to desert conditions. Ann. Bot. 2008; 101: 277–283.

16. Yang X, Zhang W, Dong M, Boubriak I, Huang Z. The achene mucilage hydrated in desert dew assists seed cells in maintaining DNA integrity: adaptive strategy of desert plant Artemisia sphaerocephala. PLoS One 2011; 6: e24346.

17. Penfield S, Meissner RC, Shoue DA, Carpita NC, Bevan MW. MYB61 is required for mucilage deposition and extrusion in the Arabidopsis seed coat. Plant Cell 2001; 13: 2777–2791.

18. Rautengarten C, Usadel B, Neumetzler L, Hartmann J, Büssis D, Altmann T. A subtilisin-like serine protease essential for mucilage release from Arabidopsis seed coats. Plant J. 2008; 54: 466–480.
19. Panikashvili D, Shi JX, Schreiber L, Aharoni A. The Arabidopsis DCR encoding a soluble BAHD acyltransferase is required for cutin polyester formation and seed hydration properties. Plant Physiol. 2009; 151: 1773-1789.
20. Thompson K, Bakker JP, Bekker RM. Soil Seed Banks of North West Europe: Methodology, Density and Longevity. 1997, Cambridge University Press; Cambridge.
21. Hendry GAF, Thompson K, Moss CJ, Edwards E, Thorpe PC. Seed persistence: a correlation between seed longevity in the soil and ortho-dihydroxyphenol concentration. Funct. Ecol. 1994; 8: 658–664.
22. Terras FR, Eggermont K, Kovaleva V, Raikhel NV, Osborn RW, Kester A. et al. Small cysteine-rich antifungal proteins from radish: their role in host defense. Plant Cell 1995; 7: 573-588.
23. De Bolle MF, Eggermont K, Duncan RE, Osborn RW, Terras FR, Broekaert WF. Cloning and characterization of two cDNA clones encoding seed-specific antimicrobial peptides from *Mirabilis jalapa* L. Plant Mol. Biol. 1995; 28: 713-721.
24. Liu Y, Luo J, Xu C, Ren F, Peng C, Wu G, Zhao J. Purification, characterization, and molecular cloning of the gene of a seed-specific antimicrobial protein from pokeweed. Plant Physiol. 2000; 122: 1015-1024.
25. Rose TL, Conceicao ADS, Jose XF, Okorokov LA, Fernandes KVS, Marty F, Marty-Mazars D, Carvalho AO, Gomes VM. Defense proteins from Vigna ungaiculata seed exudates: characterization and inhibitory activity against *Fusarium oxysporum*. Plant Soil 2006; 286: 181–191.
26. Lepiniec L, Debeaujon I, Routaboul JM, Baudry A, Pourcel L, Nesi N, Caboche M. Genetics and biochemistry of seed flavonoids. Annu. Rev. Plant Biol. 2006; 57: 405–430.
27. Smýkal P, Vernoud V, Blair MW, Soukup A, Thompson RD. The role of the testa during development and in establishment of dormancy of the legume seed. Front. Plant Sci. 2014; 5: 351.
28. Mohamed-Yasseen Y, Barringer SA, Splittstoesser WE, Costanza S. The role of seed coats in seed viability. Bot. Rev. 1994; 60: 426–439.

29. Gijzen M, Kuflu K, Qutob D, Chernys JT. A class I chitinase from soybean seed coat. J. Exp. Bot. 2001; 52: 2283-2289.
30. Schlumbaum A, Mauch F, Vögeli U, Boller T. Plant chitinases are potent inhibitors of fungal growth. Nature 1986; 324: 365 – 367.
31. Broglie K, Chet I, Holliday M, Briddle P, Knowlton S, Mauvais CJ, Broglie R. Transgenic plants with enhanced resistance to the fungal pathogen *Rhizoctonia solani*. Science 1991; 254: 1194–1197.
32. Willats WG, McCartney L, Knox JP. In-situ analysis of pectic polysaccharides in seed mucilage and at the root surface of Arabidopsis thaliana. Planta 2001; 213: 37-44.
33. Blank A, Sugiyama RH, Dekker CA. Activity staining of nucleolytic enzymes after sodium dodecyl sulfate-polyacrylamide gel electrophoresis: use of aqueous isopropanol to remove detergent from gels. Anal. Biochem. 1982; 120: 267-275.
34. Grafi G, Larkins BA. Activity of single-stranded DNA endonucleases in mung bean is associated with cell division. Plant Mol. Biol. 1995; 29: 703-710.
35. Trudel J, Asselin A. Detection of chitinase activity after polyacrylamide gel electrophoresis. Anal. Biochem. 1989; 178: 362-366.
36. Patton T, Barrett J, Brennan J, Moran N. Use of a spectrophotometric bioassay for determination of microbial sensitivity to manuka honey. J. Microbiol. Methods 2006; 64: 84-95.
37. Broekaert WF, Terras FR, Cammue BP, Osborn RW. Plant defensins: novel antimicrobial peptides as components of host defense system. Plant Physiol. 1995; 108: 1353–1358.
38. Carvalho Ade O, Gomes VM. Plant defensins and defensin-like peptides—Biological activities and biotechnological applications. Curr. Pharm. Des. 2011; 17: 4270–4293.
39. De Coninck B, Cammue BPA, Thevissen K. Modes of antifungal action and in planta functions of plant defensins and defensin-like peptides. Fung. Biol. Rev. 2013; 26: 109–120.
40. van Loon LC, van Strien EA. The families of pathogenesis-related proteins, their activities, and comparative analysis of PR-1 type proteins. Physiol. Mol. Plant Pathol. 1999; 55: 85-97.
41. Farage-Barhom S, Burd S, Sonego L, Perl-Treves R, Lers A. Expression analysis of the BFN1 nuclease gene promoter during senescence, abscission, and programmed cell death-related processes. J. Exp. Bot. 2008; 59: 3247-3258.
42. Husebye H, Chadchawan S, Winge P, Thangstad OP, Bones AM. Guard cell- and phloem

idioblast-specific expression of thioglucoside glucohydrolase 1 (myrosinase) in Arabidopsis. Plant Physiol. 2002; 128: 1180–1188.

43. Goldberg RB, Barker SJ, Perez-Grau L. Regulation of gene expression during plant embryogenesis. Cell 1989; 56: 149-160.
44. Weber H, Borisjuk L, Wobus U. Molecular physiology of legume seed development. Annu. Rev. Plant Biol. 2005; 56: 253-279.
45. Beard P, Morrow JF, Berg P. Cleavage of circular, superhelical simian virus 40 DNA to a linear duplex by S1 nuclease. J. Virol. 1973; 12: 1303-1313.
46. Benham CJ. Torsional stress and local denaturation in supercoiled DNA. Proc. Nat. Acad. Sci. USA1979; 76: 3870-3874.
47. Friedman J, Stein Z, Rushkin E. Drought tolerance of germinating seeds and young seedlings of Anastatica hierochuntica L. Oecologia 1981; 51: 400-403.
48. Hegazy AK, Kabiel HF. Significance of microhabitat heterogeneity in the spatial pattern and size-class structure of *Anastatica hierochuntica* L. Acta Oecologica 2007; 31: 332–342.
49. Western TL. The sticky tale of seed coat mucilages: production, genetics, and role in seed germination and dispersal. Seed Sci. Res. 2012; 22: 1-25.
50. Gechev TS, Hille J, Woerdenbag HJ, Benina M, Mehterov N, Toneva V, Fernie AR, Mueller-Roeber B. Natural products from resurrection plants: Potential for medical applications. Biotechnol. Adv. 2014; 36: 1091–1101.
51. Mohamed AA, Khalil AA, El-Beltagi HES. Antioxidant and antimicrobial properties of kaff maryam (*Anastatica hierochuntica*) and doum palm (*Hyphaene thebaica*). Grasas Y Aceites 2010; 61: 67-75.
52. Debeaujon I, Leon-Kloosterziel KM, Koornneef M. Influence of the testa on seed dormancy, germination, and longevity in Arabidopsis. Plant Physiol. 2000; 122: 403–413.
53. Moise JA, Han S, Gudynaite-Savitch L, Johnson DA, Miki BLA. Seed coats: structure, development, composition, and biotechnology. In Vitro Cell. Dev. Biol. Plant. 2005; 41: 620–644.
54. Coca M, Bortolotti C, Rufat M, Peñas G, Eritja R, Tharreau D, del Pozo AM, Messeguer J, San Segundo B. Transgenic rice plants expressing the antifungal AFP protein from Aspergillus giganteus show enhanced resistance to the rice blast fungus *Magnaporthe grisea*.

Plant Mol. Biol. 2004; 54: 245-259.

55. Vriens K, Cammue BP, Thevissen K. Antifungal plant defensins: mechanisms of action and production. Molecules 2014; 19: 12280-12303.
56. Hayashi T, Kaida R. Functions of xyloglucan in plant cells. Mol. Plant 2011; 4: 17-24.
57. Chen F, Nonogaki H, Bradford KJ. A gibberellin-regulated xyloglucan endotransglycosylase gene is expressed in the endosperm cap during tomato seed germination. J. Exp. Bot. 2002; 53: 215-223.
58. Voegele A, Linkies A, Müller K, Leubner-Metzger G. Members of the gibberellin receptor gene family GID1 (GIBBERELLIN INSENSITIVE DWARF1) play distinct roles during *Lepidium sativum* and Arabidopsis thaliana seed germination. J. Exp. Bot. 2011; 62: 5131-5147.
59. Grover A. Plant Chitinases: Genetic Diversity and Physiological Roles. Crit. Rev. Plant Sci. 2012; 31: 57-73.
60. Shukla P, Singh NK, Kumar D, Vijayan S, Ahmed I, Kirti PB. Expression of a pathogen-induced cysteine protease (AdCP) in tapetum results in male sterility in transgenic tobacco. Funct. Integr. Genomics 2014; 14: 307-317.
61. Balakrishnan L, Bambara RA. Flap endonuclease 1. Annu. Rev. Biochem. 2013; 82: 119-138.
62. Sugiyama M, Ito J, Aoyagi S, Fukuda H. Endonucleases. Plant Mol. Biol. 2000; 44: 387-397.
63. Givaty-Rapp Y, Yadav NS, Khan A, Grafi G. S1-type endonuclease 2 in dedifferentiating Arabidopsis protoplasts: translocation to the nucleus in senescing protoplasts is associated with de-glycosylation. PLoS One 2017; 12: e0170067.
64. Granot G, Morgenstern Y, Khan A, Rapp YG, Pesok A, Nevo E, Grafi G. Internucleosomal DNA fragmentation in wild emmer wheat is catalyzed by S1-type endonucleases translocated to the nucleus upon induction of cell death. Biochim. Biophys. Acta 2015; 1849: 239-246.
65. Giannakis IJ, Forbes PD, Zalewski PD. Ca^{2+}/Mg^{2+}-dependent nuclease: tissue distribution, relationship to inter-nucleosomal DNA fragmentation and inhibition by Zn^{2+}. Biochem. Biophys. Res. Commun. 1991; 181: 915-920.
66. Sánchez-Pons N, Vicient CM. Identification of a type I Ca^{2+}/Mg^{2+}-dependent endonuclease induced in maize cells exposed to camptothecin. BMC Plant Biol. 2013; 13: 186.

67. Gartemann KH, Kirchner O, Engemann J, Gräfen I, Eichenlaub R, Burger A. *Clavibacter michiganensis* subsp. *michiganensis*: first steps in the understanding of virulence of a Gram-positive phytopathogenic bacterium. J. Biotechnol. 2003; 106: 179-191.
68. Galiana E, Bonnet P, Conrod S, Keller H, Panabières F, Ponchet M, Poupet A, Ricci P. RNase activity prevents the growth of a fungal pathogen in tobacco leaves and increases upon induction of systemic acquired resistance with elicitin. Plant Physiol. 1997; 115: 1557-1567.
69. Hugot K, Ponchet M, Marais A, Ricci P, Galiana E. A tobacco S-like RNase inhibits hyphal elongation of plant pathogens. Mol. Plant Microbe Interact. 2002; 15: 243-250.
70. Bertini L, Caporale C, Testa M, Proietti S, Caruso C. Structural basis of the antifungal activity of wheat PR4 proteins. FEBS Lett. 2009; 583: 2865-2871.
71. Balasubramanian V, Vashisht D, Cletus J, Sakthivel N. Plant β-1,3-glucanases: their biological functions and transgenic expression against phytopathogenic fungi. Biotechnol. Lett. 2012; 34: 1983-1990.
72. Ceasar SA, Ignacimuthu S. Genetic engineering of crop plants for fungal resistance: role of antifungal genes. Biotechnol. Lett. 2012; 34: 995-1002.
73. Sharma N, Sharma KP, Gaur RK, Gupta VK. Role of chitinase in plant defense. Asian J. Biochem. 2011; 6: 29-37.
74. Miernyk JA, Johnston ML. Proteomic analysis of the testa from developing soybean seeds. J. Proteom. 2013; 89: 265-272.
75. Radulović NS, Blagojević PD, Stojanović-Radić ZZ, Stojanović NM. Antimicrobial plant metabolites: structural diversity and mechanism of action. Curr. Med. Chem. 2013; 20: 932-952.
76. Gilbert GS. Evolutionary ecology of plant diseases in natural ecosystems. Annu. Rev. Plant Pathol. 2002; 40: 13-43.

Supporting Information

Supplemental Figures S1, S2 and S3.

Fig. S1. Functional classification of released proteins from Arabidopsis (Col) seeds.

Fig. S2. Timing of release of nucleases from the *S. alba* seeds following hydration.

Fig. S3. Nuclease activities released from seed coats of various leguminous plants.

Table S1. A list of plant species used in the present study.

Table S2. Proteome parameter definition.

Supplemental data set 1. Proteome raw data of proteins released from *Arabidopsis thaliana* seeds upon hydration.

Supplemental data set 2. Proteome raw data proteins released from seed coats of *Sinapis alba* and *Anastatica hierochuntica*

Supplemental data set 3. List of proteins released from dead seed coats shared by *Sinapis alba* and *Anastatica hierochuntica.*

Legends to Figures:

Fig. 1. Nuclease activities in seed secretions of Brassicaceae species. In-gel nuclease assay demonstrating high nuclease activity in secretions from seeds of various crucifers. Seeds of *Arabidopsis thaliana* Ler and Col lines, *Capsella bursa-pastoris*, *Sysimbrium irio*, *Moricandia nitens, Diplotaxis erucoides*, *Diplotaxis harra* and *Sinapis alba* were incubated in PBS for 8 h at 4°C, the aqueous phase was collected and proteins were separated on 12% SDS/PAGE containing denatured salmon sperm DNA. Nuclease reaction was performed with or without the indicated divalent cations and activity was visualized by staining with ethidium bromide. M, protein size markers. Note that strong nuclease activity was recovered in the presence of $Mg^{2+/}Ca^{2+}$.

Fig. 2. Nucleases are released from the seed coat. A, Proteins released from seeds, seed coats and embryos of the indicated Brassicaceae species were subjected to in gel nuclease assay using denatured salmon sperm DNA as substrate. Upper panels are the EZBlue staining and the lower

panels are the nuclease assays. M, protein molecular weight markers. B, In gel RNase assay. Proteins released from seeds, seed coats and embryos of *S. alba* were subjected to in gel nuclease assay using Torula yeast RNA as substrate. Asterisks at the lower panel mark positions of active RNases. Upper panel is the EZBlue staining gel. C, In vitro endonuclease assay. Supercoiled plasmid DNA (1 μg) was incubated for the indicated time points at room temperature with *S. alba* seed secretion. Supercoiled plasmid DNA incubated with the buffer only or with 50 mM EDTA only, for 15 min, were included as controls. Lin is restriction enzyme linearized plasmid (marked by white asterisk). The positions of the different topological forms of plasmid DNA are indicated: R, relaxed form; L, linear; SC, supercoiled plasmid DNA. M, molecular size markers of 1 kb DNA ladder.

Fig. 3. Long-term survival of nucleases within the dead seeds of *A. hierochuntica*. A, Germination test of new (2015) and old (1968) seeds of *A. hierochuntica* collected from various sites. Tiran and Sanafir ecotypes refer to islands located at the straits of Tiran that separate the Red Sea from the Golf of Aqaba/Eilat. Sodom ecotype was collected near Sodom mountain and the dead sea, while Ovda ecotype was collected near Eilat. B, Dead, non viable seeds of Tiran and Sanafir store and release upon hydration active nucleases. Seeds of the indicated ecotypes were incubated in PBS for 8 h, the aqueous phase was collected and analyzed by in-gel nuclease assay using denatured salmon sperm DNA as substrate. M is the protein molecular weight markers. Long-term survival of nucleases (C) and chitinases (D) within the seed coat of *Raphanus sativus* collected at 1980. The gels in (C) and (D) were stained with EZblue following the nuclease and chitinase reactions. Asterisks in (D) indicate chitinases released from the seed coat. M, Protein molecular weight markers.

Fig. 4. The seed coat mucilage is not required for storage and secretion of nucleases. A, Ruthenium red staining of *Arabidopsis* Col seeds and *gl2* mucilage mutant. B, In gel nuclease assay for proteins released from seeds of the indicated wild type and mucilage mutant lines. M, protein molecular weight markers.

Fig. 5. Analysis of proteins released from the dead seed coats of *A. hierochuntica* and *S. alba.* A, Venn diagram showing the number of proteins recovered from the dead seed coats of each

species examined and the number of proteins shared between the indicated species. 77 proteins shared by *Anastatica* ecotypes and *S. alba* highlighted yellow. B and C, GO categorization for biological process and molecular function, respectively, of the 77 proteins shared by all species examined.

Fig. 6 Seeds of the desert plant *Anastatica hierochuntica* release antimicrobial substances. A, *Staphylococcus aureus* was grown in a flat-bottom 96-well microtiter plate in the presence of PBS, ampicillin (100 mg/L) or in the presence of substances released from embryos (*Ah embryo*) or seed coats (*Ah coat*) of *A. hierochuntica*. Bacterial growth was monitored by measuring the OD_{595} of the culture at 30 min. intervals in the course of 24 h. Each treatment was performed in triplicates and error bars represent the standard deviation. B, *A. hierochuntica* seeds released substances that inhibit conidiospore germination. Microconidia derived from *Fusarium oxysporum* f.sp. *melonis* were mixed with potato dextrose broth (PDB) alone (control) or with PDB supplemented with *A. hierochuntica* seed released substances. Mixtures were placed on depression slides and incubated in moist chamber for 24 h at 25°C in a dark and inspected under a light microscope. mc, microconidia.

Figure 1

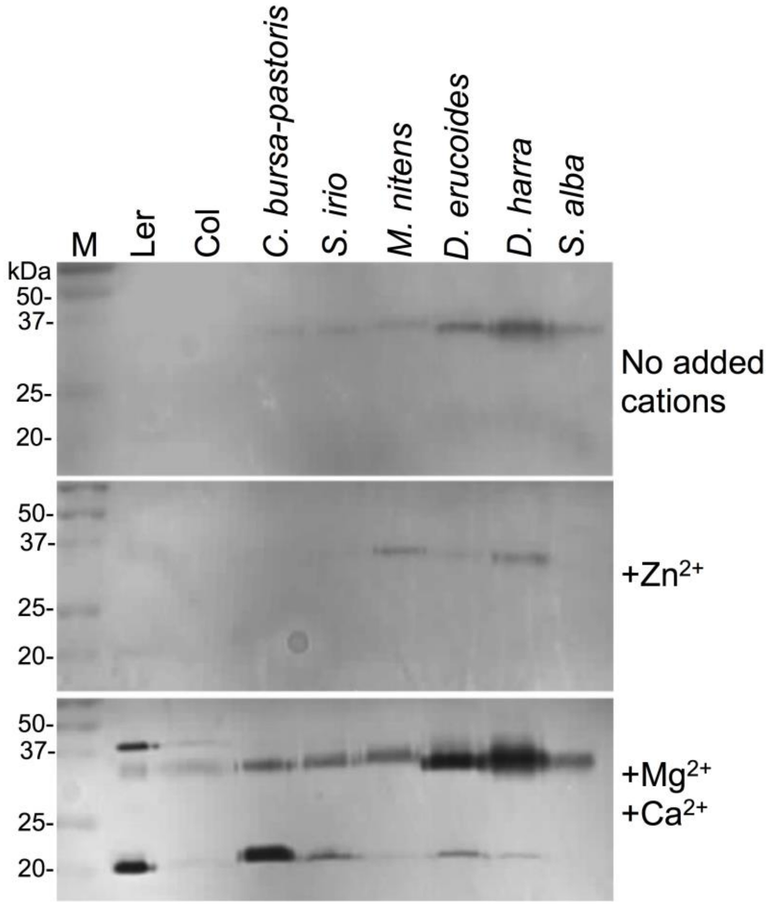

M
Ler
Col
C. bursa-pastoris
S. irio
M. nitens
D. erucoides
D. harra
S. alba
kDa
50-
37-
25-
20-
No added cations
50-
37-
25-
20-
+Zn2+
50-
37-
25-
20-
+Mg2+
+Ca2+

Figure 2

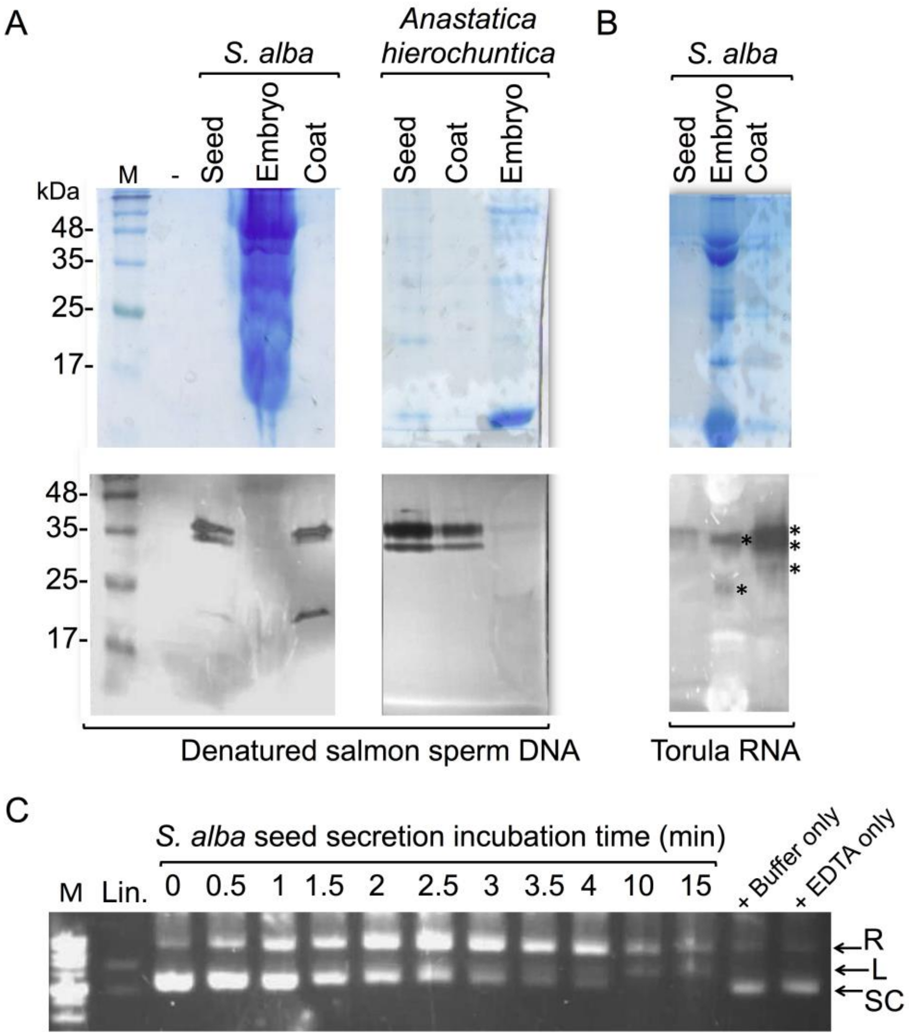
A
S. alba
Anastatica hierochuntica
M
-
Seed
Embryo
Coat
Seed
Coat
Embryo
kDa
48-
35-
25-
17-
48-
35-
25-
17-
Denatured salmon sperm DNA
B
S. alba
Seed
Embryo
Coat
Torula RNA
C
S. alba seed secretion incubation time (min)
M
Lin.
0
0.5
1
1.5
2
2.5
3
3.5
4
10
15
+ Buffer only
+ EDTA only
R
L
SC

Figure 3

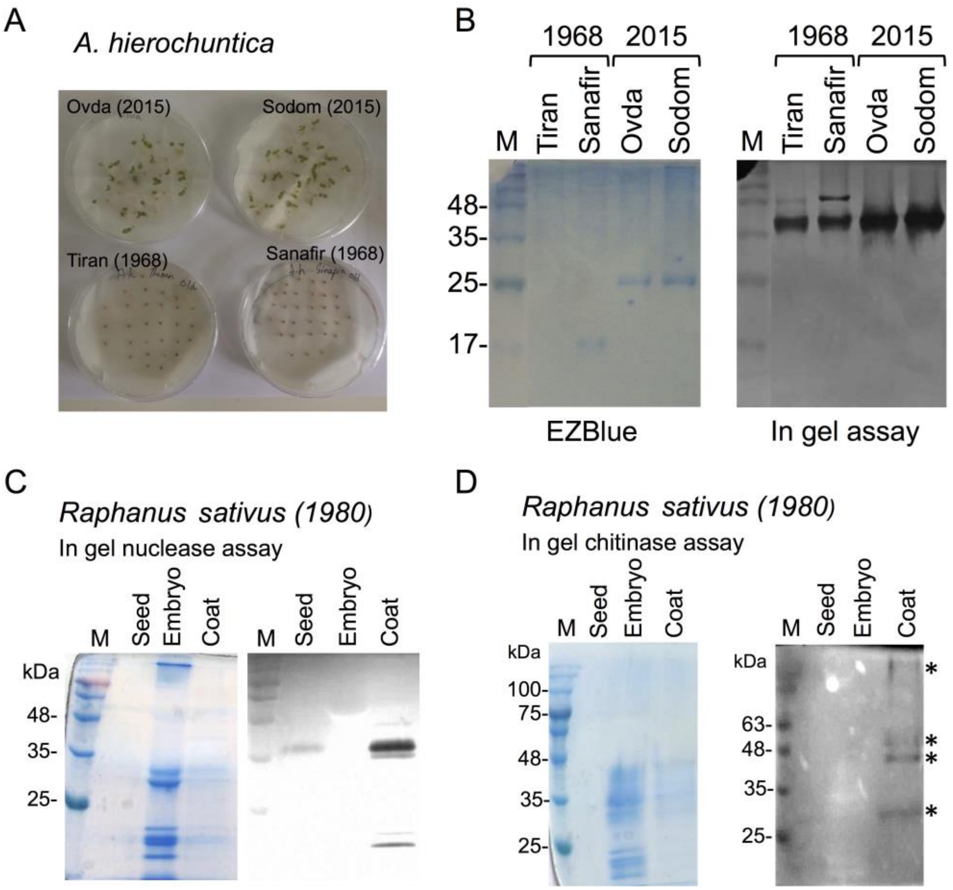

A
A. hierochuntica
Ovda (2015)
Sodom (2015)
Tiran (1968)
Sanafir (1968)
B
1968
2015
M
Tiran
Sanafir
Ovda
Sodom
48-
35-
25-
17-
EZBlue
In gel assay
C
Raphanus sativus (1980)
In gel nuclease assay
M
Seed
Embryo
Coat
kDa
48-
35-
25-
D
Raphanus sativus (1980)
In gel chitinase assay
kDa
100-
75-
48-
35-
25-
63-
*

Figure 4

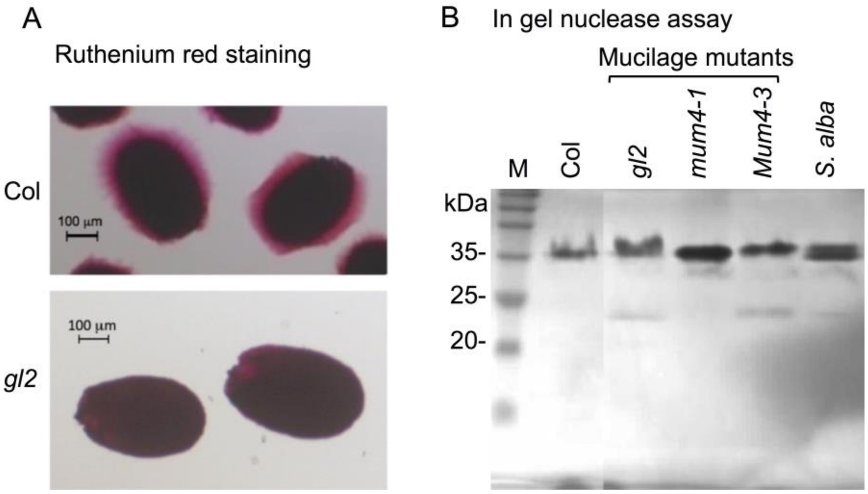
A
Ruthenium red staining
Col
100 µm
gl2
100 µm
B
In gel nuclease assay
Mucilage mutants
M
Col
gl2
mum4-1
Mum4-3
S. alba
kDa
35-
25-
20-

Figure 5

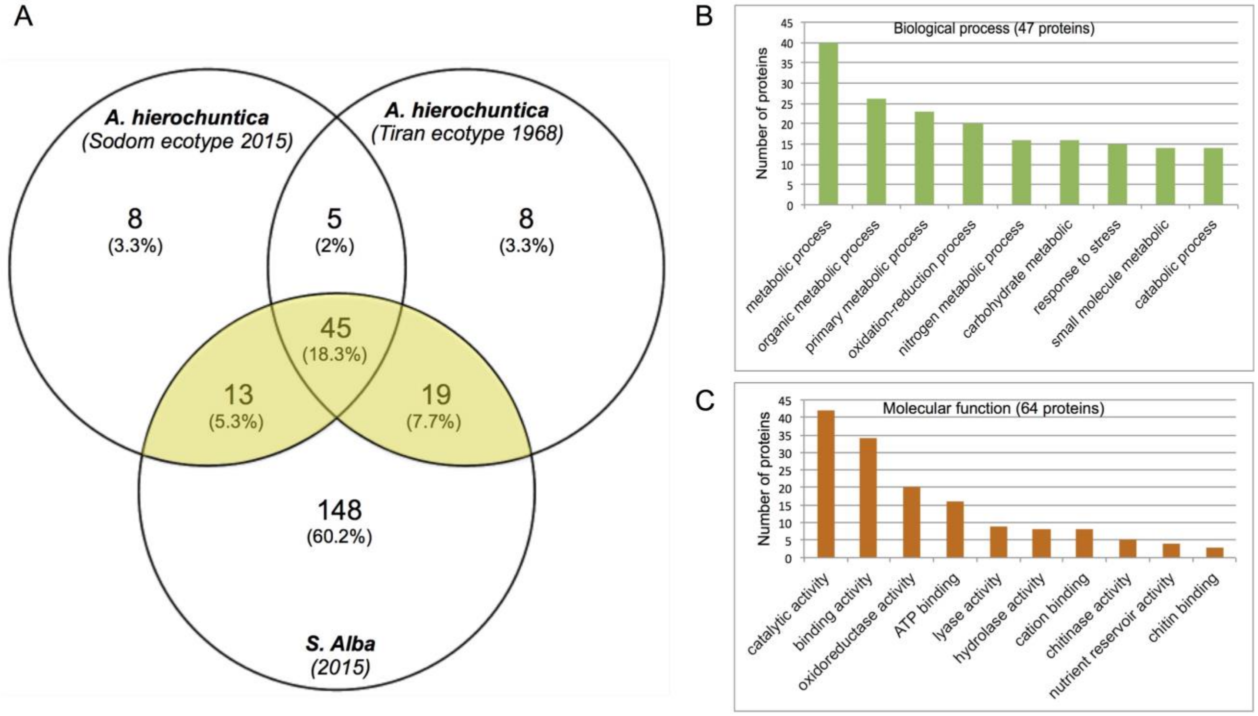
A
A. hierochuntica
(Sodom ecotype 2015)
A. hierochuntica
(Tiran ecotype 1968)
8
(3.3%)
5
(2%)
8
(3.3%)
45
(18.3%)
13
(5.3%)
19
(7.7%)
148
(60.2%)
S. Alba
(2015)
B
Biological process (47 proteins)
Number of proteins
0
5
10
15
20
25
30
35
40
45
metabolic process
organic metabolic process
primary metabolic process
oxidation-reduction process
nitrogen metabolic process
carbohydrate metabolic
response to stress
small molecule metabolic
catabolic process
C
Molecular function (64 proteins)
Number of proteins
0
5
10
15
20
25
30
35
40
45
catalytic activity
binding activity
oxidoreductase activity
ATP binding
lyase activity
hydrolase activity
cation binding
chitinase activity
nutrient reservoir activity
chitin binding

Figure 6

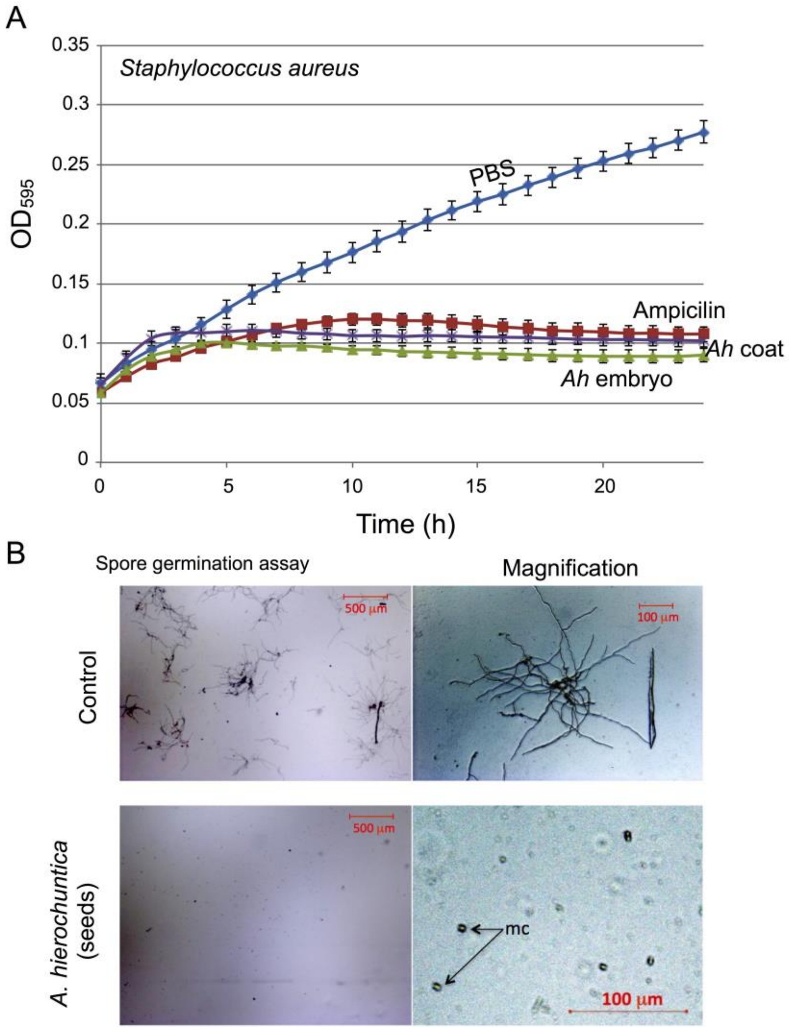

A
Staphylococcus aureus
0.35
0.3
0.25
0.2
0.15
0.1
0.05
0
OD595
PBS
Ampicilin
Ah coat
Ah embryo
0
5
10
15
20
Time (h)
B
Spore germination assay
Magnification
Control
A. hierochuntica (seeds)
500 μm
100 μm
mc

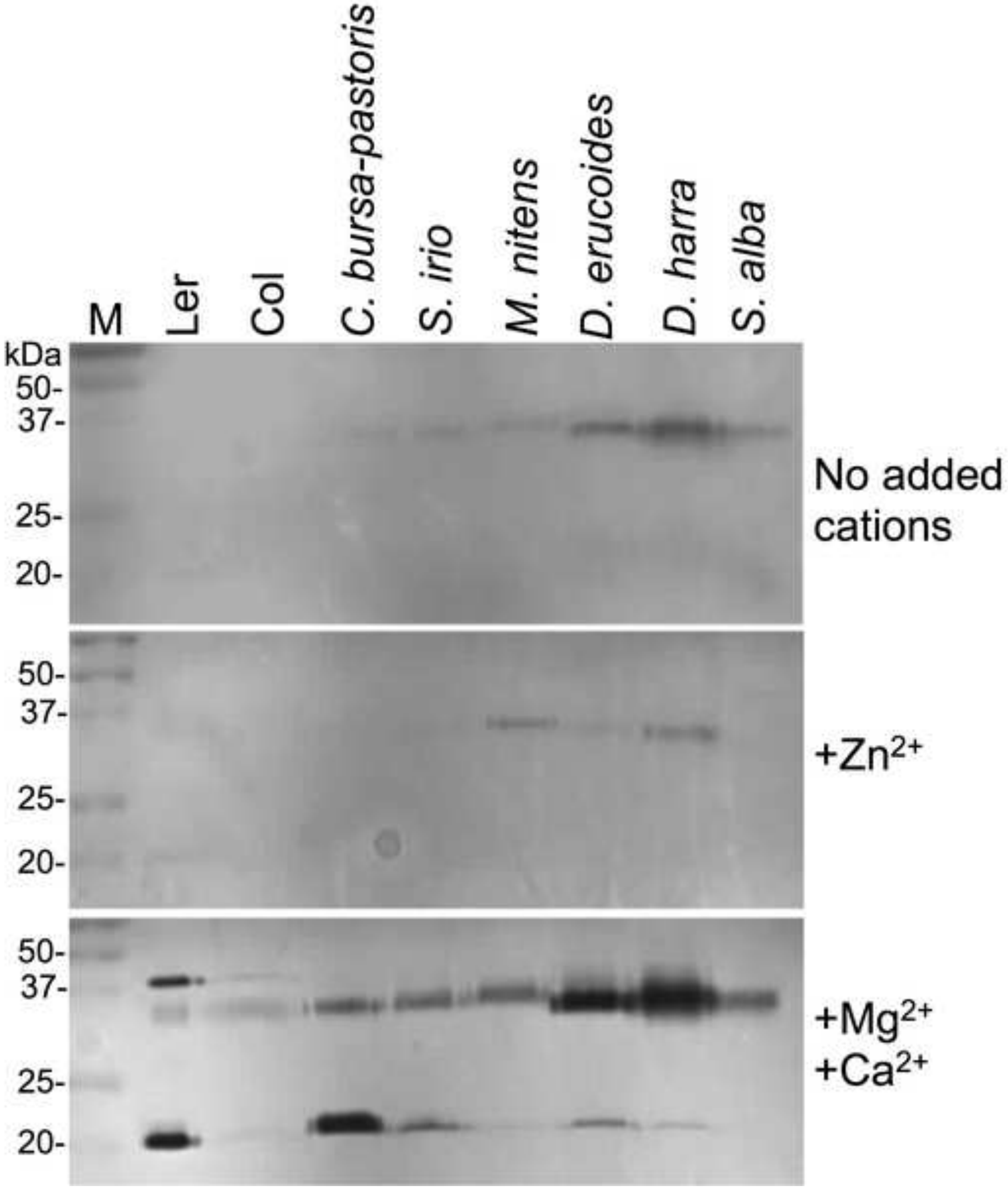
M
Ler
Col
C. bursa-pastoris
S. irio
M. nitens
D. erucoides
D. harra
S. alba
kDa
50-
37-
25-
20-
No added cations
50-
37-
25-
20-
+Zn2+
50-
37-
25-
20-
+Mg2+
+Ca2+

Figure 2

Click here to download Figure Raviv Fig. 2.jpg

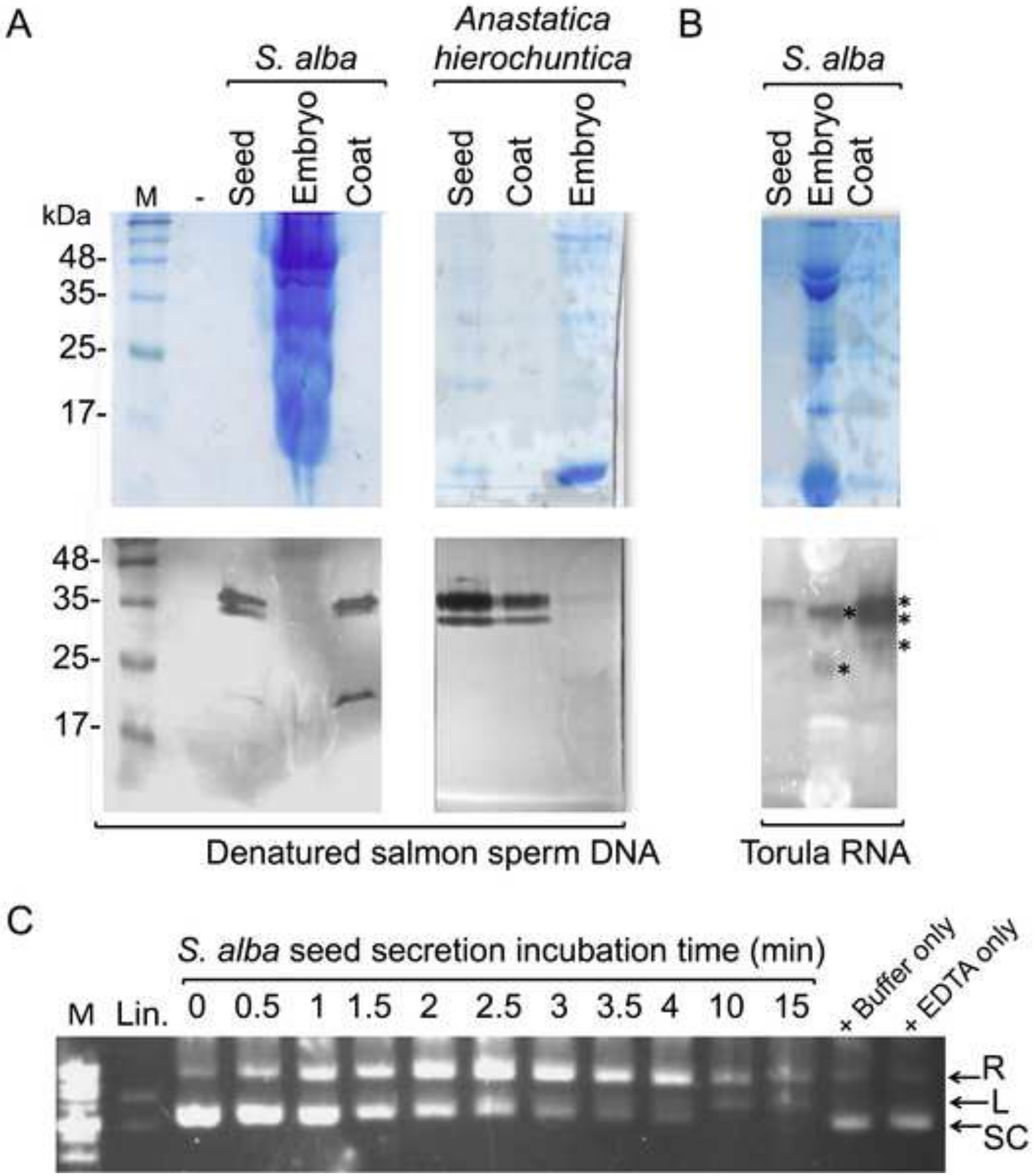

Figure 3

Click here to download Figure Raviv Fig. 3.jpg

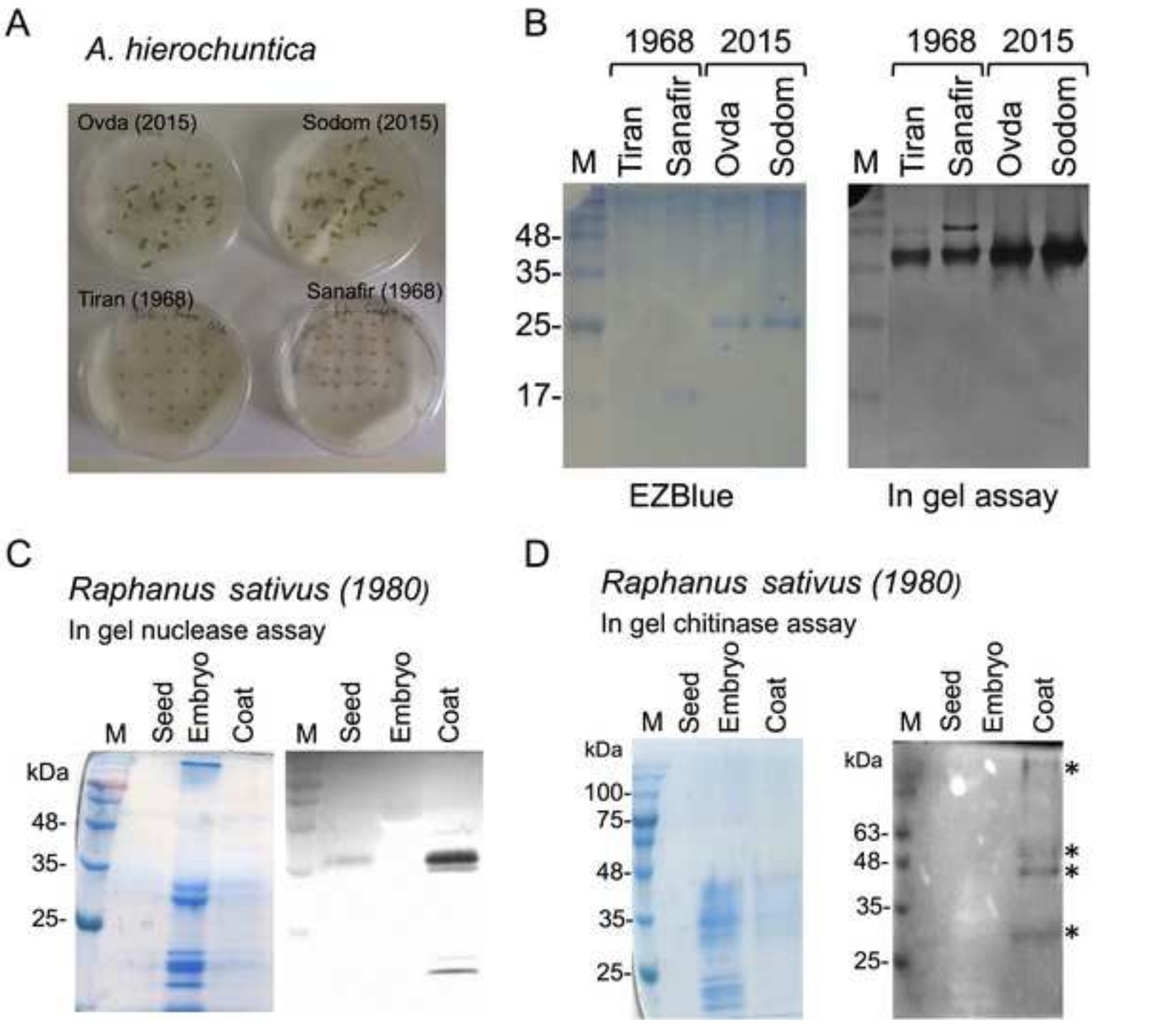

Figure 4

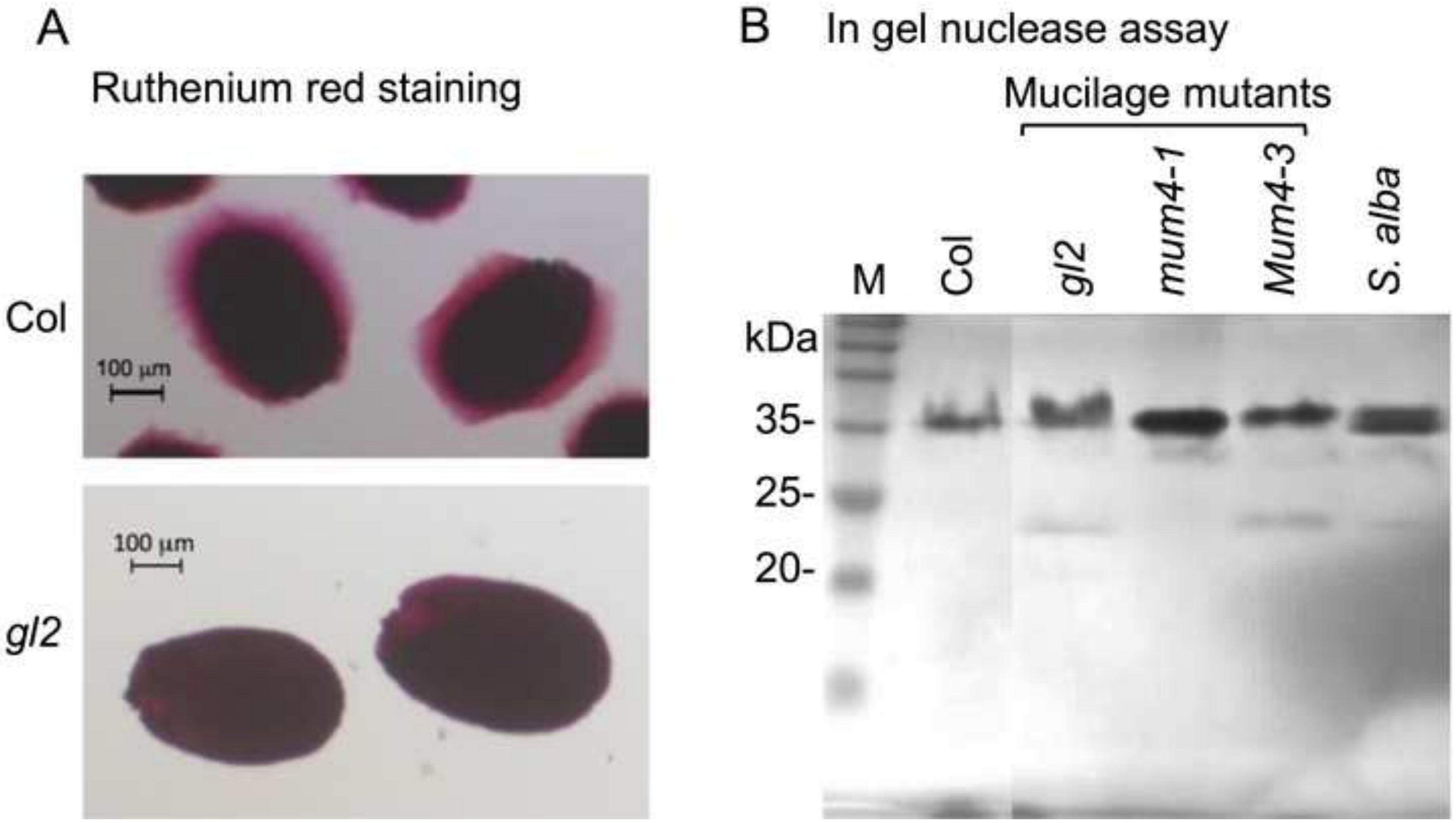
A
Ruthenium red staining
Col
100 µm
gl2
100 µm
B
In gel nuclease assay
Mucilage mutants
M
Col
gl2
mum4-1
Mum4-3
S. alba
kDa
35-
25-
20-

Figure 5

Click here to download Figure Raviv Fig. 5.jpg

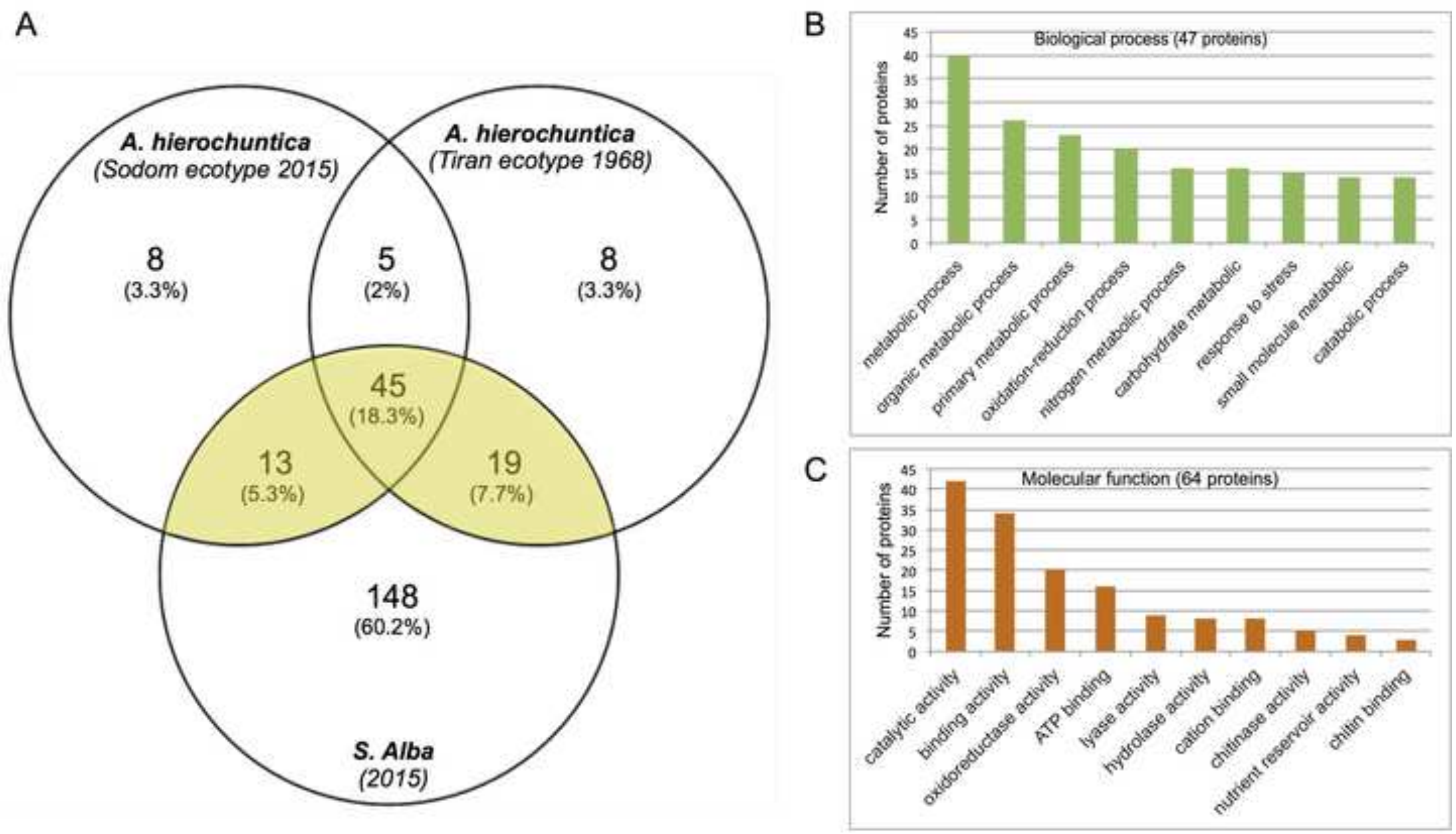

Figure 6 Click here to download Figure Raviv Fig. 6.jpg

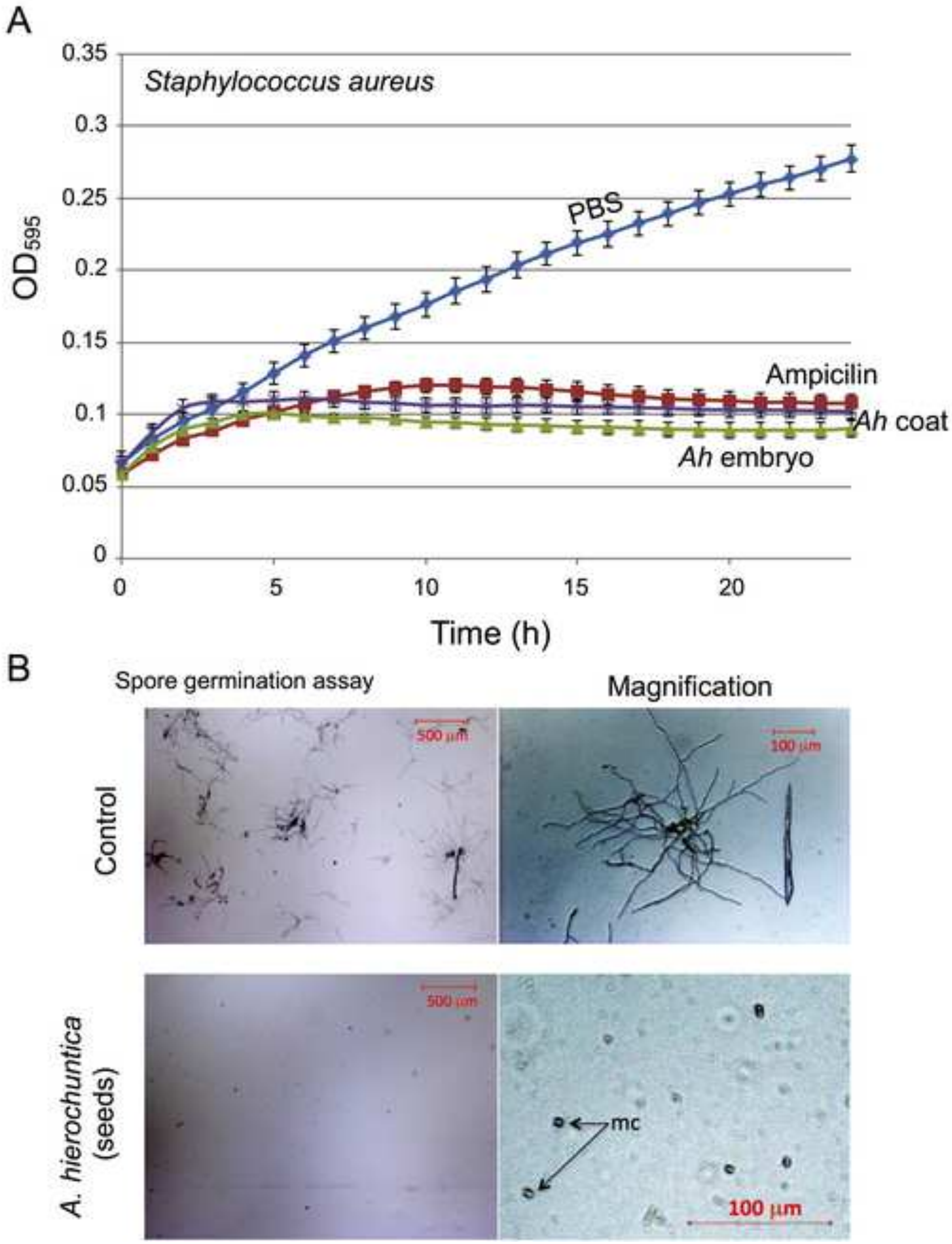

Click here to access/download
Supporting Information
Supplemental Figs S1,S2,S3.pdf

Table S1

Click here to access/download
Supporting Information
Table S1.xlsx

Table S2

Click here to access/download
Supporting Information
Table S2 Proteome parameter definition.pdf

data set 1

Click here to access/download
Supporting Information
Supplemental data set 1.xlsx

data set 2

Click here to access/download
Supporting Information
Supplemental Data Set 2.xlsx

data set 3

Click here to access/download
Supporting Information
Supplemental Data Set 3.xlsx
