# Supplementary material for: The dead, hardened floral bracts of dispersal units of wild wheat function as storage for active hydrolases and in enhancing seedling vigor
Source: PLoS One. 2017 May 11;12(5):e0177537. doi: 10.1371/journal.pone.0177537 (PMC5426743; doi:10.1371/journal.pone.0177537)
Supplement: S4 Table — (PDF) [file pone.0177537.s005.pdf]

Proteins page parameters

Feature	Description
Accession	Displays by default the unique identifier assigned to the protein by the FASTA database used to generate the report.
Description	Provides the name of the protein exclusive of the identifier that appears in the Accession column. This description appears in the table by default.
Score	Displays the protein score, which is the sum of the scores of the individual peptides.<br><br>• For Sequest results, the score is the sum of all peptide Xcorr values above the specified score threshold. The score threshold is calculated as follows:<br><br>0.8 + peptide_charge × *peptide_relevance_factor*<br><br>where peptide_relevance_factor is an advanced parameter of the SEQUEST or Sequest HT node in the “Protein Scoring Option” category with a default value of 0.4. For each spectrum, only the highest-scoring match is used.<br><br>For each spectrum and sequence, the Proteome Discoverer application uses only the highest scored peptide. When it performs a search using dynamic modifications, one spectrum might have multiple matches because of permutations of the modification site.
Score, continued	• For Mascot results, the score is:<br><br>– Standard score, which is the cumulative protein score based on summing the ion scores of the unique peptides identified for that protein. If a peptide was redundantly identified, only the highest-scoring peptide is used.<br><br>–or–<br><br>– MudPIT score, which is the sum of the “excess of ions” score over the homology or identity threshold for each spectrum plus the average threshold. For MudPIT scoring, the score for each peptide is not its absolute score but the amount that it is above the threshold. Therefore, peptides with a score below the threshold do not contribute to the score. For each peptide, the threshold is the homology threshold, if it exists; otherwise, it is the identity threshold.<br><br>By default, the Proteome Discoverer application automatically switches between the standard and the MudPIT score to calculate the protein score in the Mascot node results. It automatically uses the MudPIT score when the number of queries divided by the number of FASTA database entries

	exceeds 0.001.
Coverage	Displays by default the percentage of the protein sequence covered by identified peptides.
# Proteins	Displays the number of identified proteins in the protein group of a master protein. This number is the same as that displayed in the Protein Group Members view when you choose Search Report > Show Protein Group Members View (see this figure).
# Unique Peptides	Displays the number of peptide sequences unique to a protein group.
# Peptides	Displays the number of distinct peptide sequences in the protein group.
# PSMs	Displays the total number of identified peptide sequences (peptide spectrum matches) for the protein, including those redundantly identified.
# AAs	Shows by default the sequence length of the protein.
MW [kDa]	Displays the calculated molecular weight of the protein. The Proteome Discoverer application calculates the molecular weight without considering PTMs.<br><br>Separating proteins by molecular weight can be one of the steps in two-dimensional gel electrophoresis. You can use the protein's molecular weight as a rough constraint to estimate whether it is reasonable to identify a particular protein in a certain fraction that was searched.
calc. pI	Displays the theoretically calculated isoelectric point, which is the pH at which a particular molecule carries no net electrical charge.<br><br>The amino acids that make up proteins can be positive, negative, neutral, or polar in nature, and together they give a protein its overall charge. At a pH below their isoelectric point, proteins carry a net positive charge; at a pH above their isoelectric point, they carry a net negative charge. Gel electrophoresis can then separate proteins according to their isoelectric point (overall charge) with a polyacrylamide gel, using a technique called isoelectric focusing, which uses a pH gradient to separate proteins. Isoelectric focusing is also the first step in two-dimensional gel polyacrylamide gel electrophoresis.<br><br>When you have searched the fractions resulting from isoelectric focusing, you can use the calc. pI value to estimate whether you might expect to find a particular protein in the given fraction.
Area (in MSF files with	Displays the average area of the three unique peptides with the largest peak

isotopically labeled quantification results only)	area.
Heavy/Light (in MSF files with isotopically labeled quantification results only)	Displays the ratio of the quantification values of the heavy and light quantification channels.
Heavy/Light Count (in MSF files with isotopically labeled quantification results only)	Displays the number of peptide ratios that were actually used to calculate a particular protein ratio. For more information about ratio counts, see Ratio Count.
Heavy/Light Variability [%] (in MSF files with isotopically labeled quantification results only)	Displays the variability of the peptide ratios that were used to calculate a particular protein ratio. For more information about protein variability, see Ratio Variability.
Ratio columns (in MSF files with reporter ion quantification results only)	Display the ratio of the intensity of the fragmented tag in a sample to the intensity of the fragmented tag in the control sample.
Ratio Count columns (in MSF files with reporter ion quantification results only)	Displays the number of peptide ratios that were actually used to calculate a particular protein ratio. For more information about ratio counts, see Ratio Count.
Ratio Variability [%] columns (in MSF files with reporter ion quantification results only)	Displays the variability of the peptide ratios that showed how much confidence there is in the calculation of protein ratios. For more information about ratio variability, see Ratio Variability.
Is Master Protein	Indicates whether the protein is the master protein of a protein group. This column appears only in the Protein Group Members view when you choose Search Report > Show Protein Group Members View. For some peptides, a list of proteins might contain this peptide sequence, but none of them is a master protein. This situation can occur if the peptide contains isoleucine at a position where the master protein has leucine or vice versa.

Clicking the plus (+) sign next to any protein opens the column parameters for the associated peptides. For descriptions of these parameters, see Peptides Page Parameters.

Peptides page parameters

Feature	Description
Sequence	Displays the sequence of amino acids that compose the peptide.
# PSMs	Displays the total number of identified peptide sequences (PSMs) for the protein, including those redundantly identified.
Protein Group Accessions	Displays the unique identifiers (accessions) of all master proteins from all protein groups that include this peptide sequence.<br><br>The identifiers displayed in the Protein Group Accessions column are the same as those displayed in the Accession column on the Proteins page. They are also the same identifiers displayed when you click + to the left of a peptide on the Peptides page to display proteins.
# Proteins	Displays the number of proteins in which this peptide is found.
# Protein Groups	Displays the number of protein groups in which this peptide is found.
Modifications	Displays the static and dynamic modifications identified in the peptide.
XCorr (search-dependent)	Scores the number of fragment ions that are common to two different peptides with the same precursor mass and calculates the cross-correlation score for all candidate peptides queried from the database (Sequest searches only).
Expectation Value or Exp Value (Mascot only)	Displays the expectation value, which is a measure of the number of matches with scores equal to or better than the score values that are expected to occur only by chance. For Mascot, the smaller the expectation value is, the better the match is considered.
IonScore (Mascot only)	Refer to the Mascot documentation from Matrix Science.
Δ Score	Displays a measure of the difference between the top two scores for the peptides identified by that spectrum. The Proteome Discoverer application calculates this score as follows. For a detailed discussion of the delta score calculation, see Calculating the Delta Score.

	$$\Delta Score = \frac{\text{Score(Rank 1 Peptide)} - \text{Score(Rank N Peptide)}}{\text{Score(Rank 1 Peptide)}}$$
Rank	Displays the ordering of peptides by rank.
Search Engine Rank	Displays the original rank assigned by the search engine for all PSMs and peptide groups. For more information on this parameter, see Filtering Results by the Original Rank Assigned by the Search Engine.
# Missed Cleavages	Displays the number of cleavage sites in a peptide sequence that a cleavage reagent (enzyme) did not cleave. This number excludes cases where an amino acid such as proline inhibits the cleaving enzyme. For example, if proline resides next to lysine or arginine, trypsin does not cleave the lysine or arginine. The # of Missed Cleavages value is 0 for a complete digest of all peptides.
Charge	Displays the charge state of the peptide.
QuanResultID (in MSF files with isotopically labeled quantification results only)	Uniquely identifies a certain quantification result consisting of the quantification values for different quantification channels. For example, if you identify a peptide in both its light and heavy forms, the light and heavy peptides share the same quantification result, so the same identifier appears for both of them in the QuanResultID column.
# Missing Channels (in MSF files with isotopically labeled and area calculation quantification results only)	Displays the number of missing channels in the peptide.
# Single-Peak Channels (in MSF files with for isotopically labeled and area calculation quantification results only)	Displays the number of single-peak channels in the peptide.
Area (in MSF files with for isotopically labeled and area	Displays the area of the peak for the isotopic cluster for the peptide.

calculation quantification results only)	
Quan Channel (in MSF files with isotopically labeled quantification results only)	Displays the channel name of the peptide that is used for quantification.
Quan Info (in MSF files with quantification results only)	Indicates why a peptide was used in quantification or why it was not:<br>• No Quan Values: No isotopic or isobaric labels were detected in the sample.<br>• No Quan Labels: The peptide was not modified by a label.<br>• Redundant: The same MS/MS spectra were used for two separate peptide matches.<br>• No Proteins: The peptide is not associated with a protein, probably because it did not go above the cutoff threshold defined by the search engines, using these parameters: the Protein Relevance Threshold or Protein Relevance Factor parameter in Mascot or the Protein Relevance Threshold parameter in Sequest.<br>• Unique: The same MS/MS spectra were used for one peptide match, and the peptide belongs to one protein.<br>• Not Unique: The peptide was found in another protein.<br>• Indistinguishable Channels: The peptide does not have amino acids that could have the specified labels or it has the specified labels but has defined channels with the same delta masses, rendering the channels indistinguishable.<br>• Excluded by Method: Peptides or quantification results are excluded from protein quantification according to the Single-Peak/Missing Channels Allowed setting in the quantification method. This setting specifies the allowed number of missing quantification channels or single-peak quantification channels that can be included in valid quantification results in the ratio calculation.
Quan Info, continued	• Inconsistently Labeled: Peptides do not always display the expected amount of isotopic labeling.<br>• Filtered Out: Peptides are not used in the quantification because the applied filters removed them.<br>This column appears only when you have loaded an MSF file containing quantification results.

Quan Usage (in MSF files with quantification results only)	Indicates whether the peptide was used to quantify the protein:<br><br>•Used: The peptide was used in quantification.<br><br>•Not Used: The peptide was not used in quantification.
Heavy/Light (in MSF files with precursor ion quantification results only)	Displays the isotopic label type. This column appears only when you have loaded an MSF file containing the results of isotopically labeled quantification.
Ratio columns (in MSF files with reporter ion quantification results only)	Display the corrected ratio of the intensity of the fragmented tag in a sample to the intensity of the fragmented tag in the control sample.
Intensity	Displays the intensity of the precursor ion.
m/z [Da]	Displays the mass-to-charge ratio of the precursor ion, in daltons.
MH+ [Da]	Displays the protonated monoisotopic mass of the peptides, in daltons.
Δ M [ppm]	Displays the difference between the theoretical mass of the peptide and the experimental mass of the precursor ion.
RT [min]	Displays the retention time when the peptide was observed, in minutes.
Binomial Peptide Score	Displays a peptide score based on the cumulative binomial probability that the observed match is a random event. The value of the Binomial Peptide score heavily depends on the data scored, but usually scores above 50 indicate a good PSM.
Isoform Confidence Probability	Estimates the probability (0–100%) that the isoform is correct.
phosphoRS Site Probabilities	Estimates the probability (0–100%) of each phosphorylation site being truly phosphorylated. phosphoRS probabilities above 75% indicate that a site is truly phosphorylated.
